# Technology Development in Online Grocery Shopping—From Shopping Services to Virtual Reality, Metaverse, and Smart Devices: A Review

**DOI:** 10.3390/foods13233959

**Published:** 2024-12-08

**Authors:** Kinga Stecuła, Radosław Wolniak, Barış Aydın

**Affiliations:** 1 Department of Production Engineering, Faculty of Organization and Management, Silesian University of Technology, 44-100 Gliwice, Poland; 2 Department of Economics and Informatics, Faculty of Organization and Management, Silesian University of Technology, 44-100 Gliwice, Poland; 3 Department of Industrial Engineering, Faculty of Engineering and Natural Sciences, Manisa Celal Bayar University, Manisa 45140, Turkey; baariisaydin35@gmail.com

**Keywords:** grocery, food, online shopping, supermarkets’ service, Virtual Reality, smart devices, AI, metaverse

## Abstract

This paper presents a review of the technologies and services associated with online grocery shopping. The progress in the field of online grocery shopping has been very rapid in recent years. Hence, there was a need to systematize knowledge about the latest various solutions used in this topic. The authors searched the internet, focusing on websites of different supermarkets, shops, and other services that offer online shopping, as well as reviewed scientific papers. Based on the collected material, the authors created four thematic parts, which include: (1) supermarket services; (2) dedicated grocery delivery services and farm-to-table; (3) shopping in Virtual Reality and the metaverse; smart devices and (4) AI in food ordering—the last part includes smart devices, such as smart refrigerators, ovens, their functionality, and the services connected with them. The authors refer to 243 sources. The research includes the three following objectives: (1) exploring and presenting the emerging applied ways of online grocery shopping, (2) exploring and presenting the latest technological advances related to the digitalization of grocery shopping, (3) discussing the upcoming technologies, services, and methods in online grocery shopping. This paper provides knowledge about a wide range of solutions offered by both supermarkets and stores (e.g., shopping applications, VR applications, metaverse shopping) and other companies (e.g., deliveries, product tracking), highlighting the numerous functions available thanks to smart devices (e.g., voice control, own shopping lists, control of products, their quantities and expiration dates, management of user preferences, and many more). This paper also discusses social issues related to the presented solutions, such as their influence on consumer behavior, barriers to adoption, and the associated challenges.

## 1. Introduction

With the advancement of technology, newer and newer solutions, which are based on the Artificial Intelligence of the sensor and the ubiquitous Internet of Things (IoT), are being introduced. Thanks to technology, people are also changing their lifestyle and habits. Changes can also be observed in the field of foods, specifically grocery shopping. Just a few decades ago, it was unthinkable that food products or meals could be ordered online and delivered to the customer’s home door. While it was rational to think about online shopping for household appliances, clothes, or other everyday items, the vision of buying groceries such as dairy products, vegetables, and fruits may have sounded too futuristic. However, technology has also led to changes in this area. People can and do use various ways to purchase groceries on an increasingly large scale. Such solutions may include websites [1], smartphone applications [2], Virtual Reality (VR) [3], as well as the use of voice assistants [4] and other services based on Artificial Intelligence (AI) [5].

On the one hand, customers expect increasingly favorable solutions in the field of grocery shopping. It is obvious that a busy society often does not like to waste time shopping for groceries, and that is why it expects changes in this area [6,7]. But on the other hand, it is corporations, companies, and stores that compete in offering modern solutions to their customers. Companies compete in a harsh fight for the customer in today’s world—a world where access to products is very easy and the variety of products is huge. Companies must try to stand out with something on the market to attract customers. One of the ways is to offer customers convenience in shopping, including shopping for groceries. Currently, online grocery shopping has undergone an evolution, with supermarkets and specialized delivery services adopting innovative approaches to meet the growing demand for convenience [8].

Due to the large and rapid technological changes in the field of online shopping, the authors decided to conduct a review of the literature and the internet sources on this topic. The article concerns online grocery shopping, presenting services offered by supermarkets on their websites, dedicated grocery delivery services, solutions such as farm-to-table, and many innovative solutions that use VR, the metaverse, and AI for shopping. The research gap addressed in this article is related to the analysis of changing technology in the field of grocery shopping. This paper provides a review of how AI, VR, voice assistants, smart devices, and other modern services enhance the shopping experience. This comprehensive study offers valuable insights into the current and future state of grocery shopping technology. The article collects the latest online shopping technologies and highlights both opportunities and challenges for consumers and retailers in the grocery shopping space.

Based on the initial analysis of the literature on the latest developments in online grocery shopping, the authors found that there is a lack of articles that comprehensively review and describe a very wide range of solutions related to different possibilities of doing online shopping that are currently available. In the literature, there are articles that deal with specific solutions (we provide references in the following sections of this article), then research papers on online grocery shopping, for example [9,10,11], and just a few reviews about general online grocery shopping. The first of them is about online grocery shopping before and during the pandemic [12], another is about adoption of these kinds of shopping [13], the next one is on online grocery shopping among low-income populations and focusing on implications for policy and research [14], and the last one is on integrating internal and external factors influencing the development of online grocery shopping and its impact on shopping outcomes [15]. However, there was a lack of complex review articles that would actually collect all the major trends related to online shopping in recent times from a technological point of view. Moreover, technology is moving forward rapidly. Hence, collecting the current and up-to-date state of solutions in the topic of online grocery shopping is important for science.

The authors set three objectives of this review. These include the following:

- Ob1: Exploring and presenting emerging applied ways of online grocery shopping;
- Ob2: Exploring and presenting the latest technological advances related to the digitalization of grocery shopping;
- Ob3: Discussing the upcoming technologies, services, and methods in online grocery shopping.

## 2. Materials and Methods

This paper presents a review of different ways and technologies of online grocery shopping, focusing on the latest achievements and solutions. The authors used both literature and internet sources (in the form of websites of online stores and online services as well as articles that show and discuss specific technologies and ways of buying groceries online). In order to make a paper that contributes new knowledge to science and includes the latest and future solutions in the field of online grocery shopping, it was necessary to search the internet, because such issues are often not yet the subject of scientific articles. Therefore, the use of websites in this research was justified. The authors searched for papers in Scopus database and for websites in the Google search engine, and the keywords included “online grocery shopping”, “supermarket online grocery shopping”, “grocery online shops”, “grocery delivery”, “online supermarket”, “Virtual Reality supermarket”, “virtual stores”, “Virtual Reality grocery”, “Virtual Reality grocery shopping application”, “grocery metaverse”, “foods ordering”, “smart online grocery shopping”, “food ordering smart devices”, and “Artificial Intelligence in food ordering”. When it came to the research papers, the authors read abstracts and then reviewed the whole papers. Referring to websites, the authors familiarized themselves with their content. If they were supermarket or application websites, the authors browsed their offers and functionalities; and if they were online articles, they read them. Based on the collected material, the authors created four thematic parts. These parts are presented in the subsequent chapters in four subchapters. These parts contain the following:

- supermarket services;
- dedicated grocery delivery services and farm-to-table;
- shopping in Virtual Reality and the metaverse;
- smart devices and AI in food ordering.

The first subchapter of the next chapter describes online shopping services that are offered by supermarkets, shops, and stores. In this chapter, supermarket websites, applications, and various online shopping solutions are presented. In the second subchapter, the authors show examples of dedicated delivery services and farm-to-table technologies. These are solutions where suppliers do not have a physical store for clients, but only offer online shopping and deliver products directly to the customer. The third subchapter concerns rather futuristic solutions as they are related to various applications that allow shopping in Virtual Reality. This chapter also discusses the subject of the metaverse. Finally, the last subchapter focuses on Artificial Intelligence, smart devices, and various methods and technologies that make it easier for customers to shop online. An example could be smart refrigerators, voice assistants, automatic shopping lists, etc.

It must be mentioned that the results of the review, presented in this article, are only half of the results of a larger review regarding changes and various solutions in the field of grocery shopping (in general). The authors conducted a large review and divided the collected materials into two parts:

- in-store
- online

**Figure 1:**
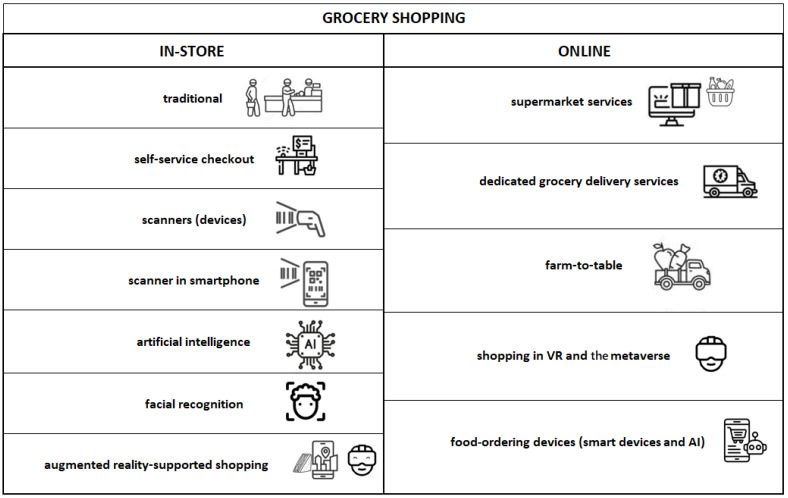
Technology in grocery shopping [authors’ own work].

Grocery shopping. Figure 1 presents this division.

The authors of this paper have already presented the first part of the review (in-store grocery shopping) in the previous paper published in [16]. However, this current article, as mentioned earlier, focuses on online grocery shopping.

## 3. Online Grocery Shopping

### 3.1. Supermarket Services

Innovations in the field of grocery shopping are not limited only to physical stores; they have also been significantly expanded to the online realm. While it was once common for consumers to use the internet primarily for purchasing items like clothes, electronics, and other durable goods, the idea of buying everyday essentials such as potatoes and lettuce online would have seemed unthinkable just a few decades ago. However, today, ordering fresh produce, dairy, and even specialty food items online has become an integral part of daily life. This shift not only highlights the growing consumer trust in online platforms, but also underscores the convenience and efficiency that digital solutions bring to the grocery shopping experience. It turns out that people have become so fond of the convenience of online shopping that they are increasingly opting to do their grocery shopping online as well. The ease of browsing, selecting items from the comfort of home, and having groceries delivered directly to their doorstep has made online grocery shopping an appealing option for many consumers.

Supermarkets have adapted to these changes by offering a range of online shopping services that cater to the needs of modern consumers. With the growing demand for convenience and efficiency, online grocery shopping has become increasingly popular. Popular supermarkets and major retailers have developed sophisticated online platforms and mobile applications to meet these demands, providing customers with a seamless shopping experience. The major supermarket chains have invested heavily in creating user-friendly online platforms and mobile applications that enable consumers to browse, select, and purchase groceries from the comfort of their homes. These platforms offer a wide array of features designed to enhance the shopping experience. For example, users can search for products by category, brand, or dietary preference, compare prices, and view detailed product descriptions and nutritional information.

Supermarkets like Carrefour [17], Tesco [18], Intermarche [19], Auchan [20], Selgros [21], El Corte Ingles [22], Biedronka [23], and Leclerc [24] have tailored their online services to accommodate different shopping preferences. Supermarkets have developed mobile applications that allow customers to easily navigate their product offerings, create shopping lists, and track their order history. Their online platforms provide a complete shopping experience, where consumers can explore weekly promotions, use digital coupons, and access personalized product recommendations based on their shopping habits.

Supermarkets offer the possibility of making online purchases through their websites, but many supermarkets have also introduced their mobile applications. On the website and in the application, you can select products, add them to the basket, remove them from the basket, use promotions, and choose the time and place of delivery. It is worth emphasizing that the mobile application will certainly be a slightly more convenient solution than the website due to the fact that today’s customers have their mobile phone at hand 24 h a day, thanks to which they can constantly control their purchases, make changes, and also track their shipment on an ongoing basis.

One of the key advantages of online grocery shopping is the flexibility it offers in terms of order fulfillment. Supermarkets provide customers with several options to receive their groceries, making it easier for them to integrate shopping into their daily routines. For example, customers can choose, among others:

- home delivery;
- the “Click & Collect” option.

When it comes to home delivery, customers can select a preferred delivery time, ensuring that their groceries arrive when it is most convenient for them. These services often include the option to track delivery in real time, providing customers with updates on the status of their orders.

The “Click & Collect” service allows customers to place their orders online and then pick them up at a designated store location. This option is ideal for shoppers who prefer to avoid delivery fees or need to collect their groceries quickly. Upon arrival at the store, customers can simply collect their pre-packed orders without having to spend time browsing aisles. Some stores even offer drive-thru pickup options, where staff members bring the groceries directly to the customer’s vehicle.

It is also worth mentioning that the option of online shopping is also convenient due to the fact that in some applications, customers can create their permanent shopping list. In addition, they can plan the delivery of purchases in advance without worrying about forgetting something. The customer, thanks to online shopping, and especially in the application, which is always at hand, has unlimited time to think about their purchases.

The common usage of supermarket online grocery services can influence people differently—positively and negatively. Examples of social impact can be as follows:

- the improved work-life balance by saving time, reducing the need for physical store visits, and providing more flexibility in daily routines;
- benefits for elderly and disabled people by providing easier access to groceries without the need for travel;
- more satisfied consumers through personalized shopping experience; adjusted promotions, recommendations, and shopping lists enhance consumer satisfaction.
- decrease in community engagement and social interactions that occur in physical stores; people can become more and more closed and isolated;
- the risk that society will become more demanding and choosier toward products due to online shopping, which provides anonymity or half-anonymity;
- job and duties shift (cashiers, salespeople, technical people, etc., will have to adjust to new duties connected with the online service) and connected with it resistance to changing positions, stress, pressure, and lack of ability to adapt;
- data privacy problems and concerns; increased collection of consumer data; potential misuse of personal information;
- the risk of widening the digital divide for those less comfortable with technology;
- a challenge for smaller local grocery stores that cannot compete technologically with large supermarkets and other online services;
- challenges in service evaluation (online platforms enable anonymous or semi-anonymous reviews, increasing the likelihood of negative feedback; this anonymity can lead to harsher criticism than in face-to-face interactions).

### 3.2. Dedicated Grocery Delivery Services and Farm-to-Table

**Figure 2:**
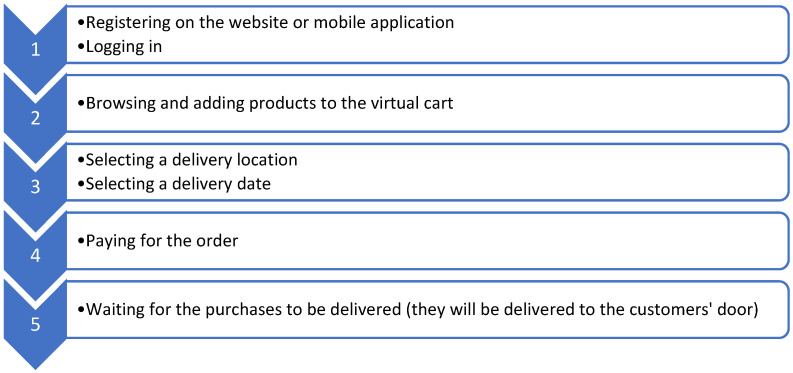
The scheme of online grocery shopping [author’s own work].

Dedicated grocery delivery services have emerged as a convenient alternative to traditional grocery shopping, operating exclusively online without physical storefronts. These online-only platforms allow customers to order a wide range of grocery items from the comfort of their homes, with goods delivered directly to their doorsteps. These services often provide user-friendly apps and websites, personalized recommendations, and subscription options for regular deliveries, catering to the growing demand for convenient and time-saving shopping solutions. Figure 2 shows the scheme of online shopping. To be able to purchase groceries online, the client must register and then log in to the website of a given store or use a mobile application for a smartphone. The website or app then offers a wide range of food products, most often organized into different categories. Various promotions and discounts are often displayed. After viewing the products, the client can easily add them to the virtual shopping cart. Once the customer has finished adding products to the virtual cart, their purchases are summarized. Then they must select the place and day of delivery of the purchases. And often, even some stores offer the opportunity to choose a specific delivery hour. After paying the bill, all the client has to do is wait for the products to be delivered to their door.

In 2007 Amazon started a grocery delivery service named Amazon Fresh, as can be read in the paper from Sundarabharathi [25]. The company established an online food delivery service in a few areas. Currently, according to the authors, in addition to four locations in Germany, Amazon Fresh presently delivers to 19 major US cities. In the literature, there is some research on this service. For example Rana et al. [26] analyzed customers’ satisfaction with the Amazon Fresh service. They examined e-grocery customer reviews downloaded from the Amazon Fresh website. They used text mining and machine learning (ML) models. The study concluded that a significant majority of customers were satisfied with their online grocery purchases from Amazon Fresh, citing the quality of products, timeliness of delivery, and overall convenience of the shopping experience. Additionally, many customers expressed a high likelihood of recommending Amazon Fresh to others, particularly due to the excellent customer service and reliable delivery. In another article [27], the authors also came to very positive conclusions about the Amazon Fresh service. The authors studied customer satisfaction in India. Compared to other stores in India, Amazon lecture very well. In another paper, the authors [28] identified effective strategies used by Amazon Fresh in logistics, organization, and marketing. According to the authors, these practices could be valuable for other countries’ online grocery stores to implement as Amazon Fresh should be a role model when it comes to online grocery shopping.

Another example of an online grocery shopping service is Instacart [29]. It is a leading grocery delivery service in North America, founded in 2012. The platform allows customers to order groceries online and have them delivered to their doorstep in as little as an hour. Instacart’s business model leverages a network of personal shoppers who pick, pack, and deliver orders, providing a convenient and flexible shopping experience [30]. The service has grown rapidly, especially during the COVID-19 pandemic, as more consumers turned to online shopping for safety and convenience [31,32]. Instacart continues to innovate with features like contactless delivery, real-time order tracking, and partnerships with retailers to expand its reach and improve the customer experience. It is also worth noting that the company has invested a very highly developed and advanced database for product search and tracking, also using Artificial Intelligence and learning algorithms [33,34]. Such solutions provide the company with a competitive advantage and contribute to high satisfaction from customers who can efficiently use the website and the application and get personalized item recommendations [34]. When it comes to Instacart, some other authors [6] conducted a study that compared online and offline grocery shopping behaviors using data from brick-and-mortar stores and Instacart online shopping. It found that online shopping baskets have a lower variety of food categories and items purchased compared to offline trips. Instacart baskets also showed a higher similarity between successive trips within households, indicating potential consumer inertia and stronger brand loyalty. Moreover, Instacart trips tended to include fewer fresh vegetables and impulse purchases like candy and snacks, which may impact competition dynamics and consumer behavior in online grocery markets. These findings underscore the challenges for new entrants in establishing customer loyalty and highlight implications for retailers and online platforms in managing product offerings and consumer engagement strategies.

There are more examples of online grocery shops. For example, there is Peapod—an American delivery service from Chicago that has been in operation since 1989 [35]. In America, there is also FreshDirect, which has offered grocery products since 2002 [36]. They provide grocery delivery to the greater New York City metropolitan area, with seasonal service to eastern Long Island and the Jersey Shore.

There are also such services offered in Europe. In Poland, a popular company is Frisco.pl, which was founded in 2006 [37]. Currently, it offers over 8000 products online. The company has eight warehouse locations in eight large Polish cities (Warsaw, Krakow, Poznań, Katowice, Wrocław, Gdańsk, Gdynia, Sopot), but it also delivers purchases to nearby cities. Frisco.pl leverages advanced logistics to ensure efficient and reliable delivery services. According to [38], Frisco.pl’s logistics network incorporates real-time inventory management and sophisticated routing algorithms to minimize delivery times and ensure product freshness. Frisco.pl’s robust logistics infrastructure enables them to serve not only urban centers but also nearby towns, maintaining high standards of service quality and customer satisfaction across a wide geographical area.

The Netherlands offers a service called Picnic, which, as it presents on their website, is a “supermarket on wheels” [39]. In the literature, Baarsma and Groenewegen [40] presents research on online shopping in the Netherlands based on Picnic. The research demonstrates a substantial increase in online grocery shopping demand in the Netherlands due to COVID-19, influenced by both local and national pandemic conditions. Data from the Dutch online supermarket Picnic reveals that local hospital admissions boosted app traffic by 7.3%, with a smaller yet significant impact on sales per order. This spike in online activity suggests a behavioral shift towards online shopping, driven by the need to minimize infection risk. Growth metrics from 2018 and 2019 indicate that the pandemic accelerated an already growing trend, though the exact contribution of COVID-19 to this growth remains complex to quantify. Post-pandemic projections (supported by a Nielsen survey) suggest that many consumers will likely continue shopping for groceries online. These findings imply that governments should promote online shopping to reduce transmission risks during the pandemic, while also ensuring broad access to fast internet and digital literacy programs to facilitate long-term adoption of e-commerce.

There are more examples of such online grocery shops around the world. There is Ocado in the UK, which was founded in 2000 [41]. The leader in the Swiss market among online supermarkets is Migors Online [42]. Australian people can buy groceries online in Woolworths [43]. Jumia operates in multiple African countries, offering an online marketplace that includes groceries among other products [44]. On Jumia’s website, one can read that it is the leading pan-African e-commerce platform active across 11 countries in the continent. Its platform consists of a marketplace connecting thousands of sellers to millions of consumers, with integrated logistics and digital payment services. In India, one of the biggest online supermarkets is BigBasket [45]. It serves customers in major cities with a wide selection of products. In 2016 Freshippo, a retail chain for groceries and fresh goods in China, was launched [46]. What must be highlighted is that Freshippo’s proprietary fulfillment system enables 30-min delivery to customers living within a three-kilometer radius of a Freshippo store.

According to MediaRadar Blog [47], consumers increasingly embrace online grocery shopping. Consumer behavior is shifting as more people get used to the ease and convenience of ordering groceries online. Clients’ motivations can differ—they can replenish household staples or pick up dinner ingredients. But in every case, online delivery services offer them speed and simplicity. Moreover, customers can order from a website or smartphone and have their items arrive in less than an hour without driving to the store or waiting in checkout lines. The pandemic accelerated the adoption of grocery delivery, and this trend appears to go up. It was also noted that once consumers try such services, they often become ongoing customers.

**Figure 3:**
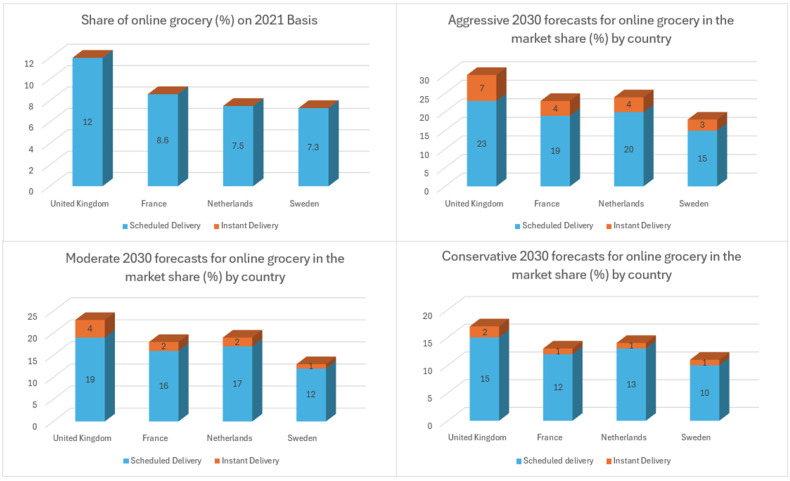
Share of online grocery in the food-at-home market, % by leading countries. Source: own work based on: Source: [48,49].

**Figure 4:**
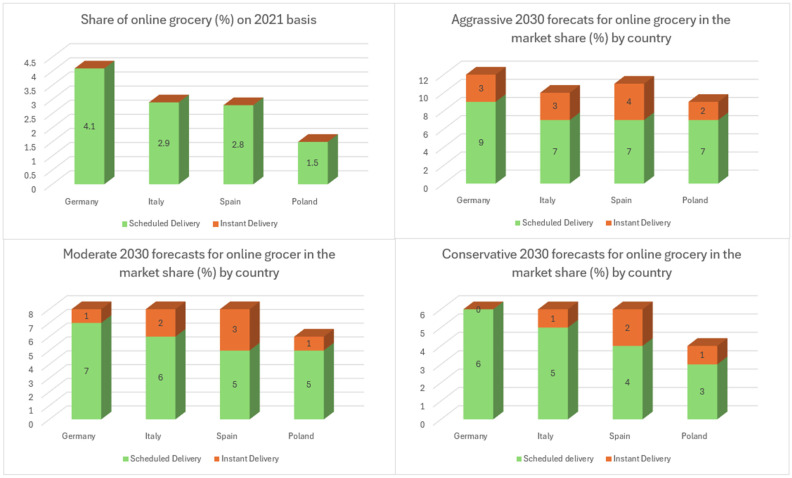
Share of online grocery in the food-at-home market, % by catching-up countries. Source: own work based on: [48,49].

People quickly adapted to online channels, attracted by their greater convenience, changing their behaviors and expectations. This shift also reshaped the competitive market landscape, with new players entering the market, often supported by significant investments. A McKinsey survey [48,49] of European consumers shows that most respondents plan to use online grocery services nearly as often as in 2021. However, the results vary significantly by country; consumers in advanced online markets plan to shop more online. Their survey included chosen European countries as leading countries (the United Kingdom, France, the Netherlands, and Sweden) and is still catching up with countries (Germany, Italy, Spain, and Poland), as shown in Figure 3 and Figure 4.

In leading countries [48,49], online grocery could comprise 18 to 30 percent of the food-at-home market by 2030 in the aggressive scenario. Scheduled delivery, promising same-day service, will account for most of this share. Instant delivery, defined as delivery on demand with a 15- to 30-min lead time, could reach 3 to 7 percent of the total food-at-home market in these countries. Researchers anticipate that online grocery will continue to expand through 2030, driven by several factors expected to influence demand. With online pure competitors disrupting markets, traditional grocery retailers must now identify key value propositions to focus on, establish a scalable operating model, and consider partnerships to enhance their existing strengths; in this case, understanding the sources of future growth is highly crucial.

First, evolving customer behavior is helpful as online grocery reaches new segments. Previously, online shopping primarily attracted young, urban, affluent families looking for the convenience of home delivery for large baskets. Today, online offerings have broadened to cater to various shopping needs and customer groups. For instance, click-and-collect models have started to be popular. All in all, countries that have been slower to adopt new trends will lose their market share.

Moreover, rising investment and competition are essential. The development of the online market has caught a high level of investment. Players must determine how to compete in the market and where to invest their resources. The online market structure is still making progress, and the future state of the market will likely reflect current offline propositions, either replacing or enhancing them. For example, full-basket offerings are similar to supermarkets, and instant delivery serves as the online equivalent of a convenience store and a small supermarket. Also, some companies provide lower minimum order requirements and free delivery, focusing on value for money in their product pricing. However, customers often must accept compromises in various products, delivery choices, and additional services.

Finally, technology is changing several aspects of the online grocery value chain, from user experience and order preparation to last-mile delivery. Developments in technology could make currently unprofitable business operations sustainable in the next years. As an example, increasing personalization for each customer could boost order sizes for large-basket deliveries, while automation could reduce costs for order preparation and delivery. In the long term, these technological advancements might make online grocery operations cheaper than physical stores, allowing grocers to offer lower prices online. This shift could reduce the advantage of physical retail, attracting new consumer segments, such as value seekers, and potentially causing significant growth in the online grocery market.

Traditional retailers that are not active in the online market risk losing market power in the future. Investigators have to take multiple actions. Grocers should view online as a critical element of future growth and invest in fulfillment, last-mile delivery, technology, and talent, involving developing a new approach that unites new capabilities and methods with existing strengths. Evaluating the effect of online growth on physical stores is another essential point. The rise of online shopping will significantly impact physical stores; offline incumbents must establish store networks to reconsider their overall strategies. As sales volumes shift online, grocery stores may need to downsize their physical spaces and minimize costs. To achieve profitability, businesses must be ready to implement targeted changes to make unprofitable ventures profitable. Established companies can leverage their scale, brand recognition, and existing infrastructure. Retailers who act decisively and invest strategically now will be well-positioned to secure a successful and sustainable market position.

Another aspect of grocery shopping is ordering and buying vegetables and fruits. Products produced on farms are our most essential food sources: meat, milk, eggs, vegetables, fruits, and many grains. It is crucial to access farm products online or offline, examine potential issues, and see how farmers adapt to new sales methods. In many countries, most food, including organic fruits and vegetables, is sold through supply chains managed by large wholesalers and supermarket chains. Additionally, some food is distributed through local marketing channels such as specialty stores, food box schemes, farmers’ markets, and community-supported agriculture [50]. The development of internet technology has expanded trading channels for fresh agricultural products and lowered trading barriers. This increased competitiveness has created more opportunities for the industry. However, challenges still need to be addressed, such as distribution difficulties, fragmented supplies, high platform operating costs, unequal product information, and low market supervision [51]. Farmers participating in online sales platforms may face interruptions due to internal and external factors. The village environment significantly impacts both large- and small-scale farmers. The researchers suggest developing the village environment to support e-commerce development and laying a solid foundation for its growth. Formal and informal financial institutions should prioritize building infrastructure to support farmers’ short- and long-term investments. Most importantly, farmers should recognize the importance of digital transformation in the agricultural value chain [52]. Surveys indicate that farmers aim to sell their products directly to consumers through the shortest possible ways. The most commonly used ways in a short supply chain are on-farm sales and telephone orders. However, despite their potential, farms rarely sell their products at farmers’ markets, celebrations, anniversaries, or e-shops. Farmers prioritize the product’s quality, freshness, locality, customer recommendations, and loyalty when selling their products. Based on the findings, academics propose enhancing sales support through new marketing approaches for demanded products, such as locally fresh products produced on family farms [53]. Blockchain-based farming marketplaces are essential for enabling trade between farmers and third-party stakeholders, such as food-processing companies and retailers. Researchers developed a Blockchain-based marketplace named “FarMarketplace”, which is integrated into a broader ecosystem called “FarMarket”. The study also outlined intelligent contract templates for transactions involving farmers, third-party consumers, and deliverers [54].

The COVID-19 pandemic led to increased direct marketing from farmers using online sales and marketing—understanding strategies for navigating various crises, from wildfires to pandemics. Online technologies can help farmers maintain their customer connections and sales when other market channels are disrupted. However, many farmers lack the resources needed to utilize technology effectively. Findings indicate that common barriers include a lack of knowledge, labor, and reliable internet access [55]. From a customer’s perspective, product quality, logistics service quality, online word of mouth, and website information quality significantly impact experienced and potential consumers. However, website information quality does not considerably affect potential consumers [56]. Additionally, health awareness, price factors, convenience, familiarity with the online shopping process, and the amount of money spent on agricultural products all significantly impact consumers to varying degrees. These findings can offer valuable insights for agricultural e-commerce enterprises, helping them adapt to consumer demand from a marketing perspective. Adapting is crucial for capturing market share and boosting agricultural e-commerce sales [57]. Consumers with scheduling and availability constraints that limit them from attending weekly farmers’ markets can still buy food directly from farmers through online platforms. Additionally, the availability of products online may lead to increased competition, which can result in lower prices and a wider variety of products [58]. As we can see, from farmers’ and consumers’ perspectives, some areas need further research for farm-to-table ideas.

Last but not least, challenges for developing grocery delivery services must be mentioned:

- ensuring timely and accurate deliveries;
- a need for complex logistics;
- a need for real-time inventory management;
- a need for providing optimized routing for deliveries;
- high operating costs;
- maintaining a fleet of delivery vehicles;
- managing warehouses;
- dynamic management;
- quality control (demand for groceries);
- meeting consumer demands for faster delivery times;
- challenge of breaking through in the market;
- cybersecurity risks (sensitive customer data, such as payment information, and personal data);
- a need for strong security measures;
- anonymous or semi-anonymous customer reviews (they can harm reputations).

### 3.3. Shopping in Virtual Reality and the Metaverse

When it comes to online shopping, there is also another innovative way to browse online supermarkets to find products. This solution can be a Virtual Reality. Although Virtual Reality is usually used for entertainment [59,60], training [61], education [62], and marketing [63], it should be noted that the area of virtual shopping is also very developing. There are already solutions in which customers can visit stores using VR goggles. They can enter a fully digital grocery store environment from the comfort of their homes. Wearing a Virtual Reality headset, shoppers can navigate virtual aisles, pick up and examine products, and even see product details just as they would in a physical store. This solution eliminates the need to physically visit a store, saving time and effort. It makes shopping comfortable and more adaptable to the needs of clients as online shopping via website or application cannot be as immersive as a VR experience. The topic of grocery shopping using VR is described by both practice and literature.

The first practical example is a service by Tesco, which has been developing 3D e-commerce offerings. In 2012 they created a 3D virtual store that shoppers could walk through via their smart TVs [64]. Clients could use motion sensor controls to walk into the virtual store and buy products. They just needed to touch a given product on the shelf virtually.

Tesco in South Korea introduced innovative virtual shops, though not directly connected with Virtual Reality. Tesco innovatively addressed the challenge of South Koreans’ long working hours by introducing “virtual stores” in metro stations and bus stops, displaying products on walls [65]. Commuters can scan the QR code of the selected product via the Tesco Homeplus application on their smartphones, and the product will then be delivered directly to their homes at selected times [66]. This allowed busy individuals to place grocery orders while waiting for trains or buses, effectively turning a busy lifestyle into a convenient shopping opportunity and creating a new market adjusted to the country’s demanding work culture.

Another example is a service called HappyReality developed by HappyFresh, which is the first and fastest-growing online grocery platform in Southeast Asia and was founded in 2014 [67]. HappyReality allows customers to experience Virtual Reality grocery shopping using VR goggles [68]. It helps replicate the most realistic images, sounds, and other sensations from a real-world grocery shopping experience, giving users the ultimate best of shopping. This solution has just been prototyped; however, the company plans to develop this functionality in the future to let clients do their shopping online in VR. HappyReality has a heat vision function that clients can activate to identify their favorite brands in seconds. It will even help customers spot the most popular products in any category, in case they don’t know which item to buy.

Amazon introduced retail innovation in shopping malls in the form of VR kiosks [69]. This service was available for customers to promote Prime Day. Clients could transfer themselves to the city filled with Prime Day products. Utilizing Oculus Rift and Oculus Touch controllers, customers can interact with products in a highly detailed 3D environment, examining different items, including grocery products. This experience aims to blend the convenience of online shopping with the sensory engagement of physical retail, potentially transforming how consumers interact with products. This service is planned to be developed.

The study of Ketoma et al. [70] developed and validated a VR grocery shopping application. First, it investigated navigation methods within the virtual store, integrating full-body tracking through multiple Microsoft Kinect v2 sensors to enable natural movement using a walking-in-place approach. Second, it conducted a qualitative usability evaluation, confirming important features for remote grocery shopping and identifying specific requirements unique to VR grocery shopping. According to the authors, VR grocery shopping addresses some of the limitations of regular online grocery shopping. However, there are still some challenges, primarily delivery, product quality, and service quality. Some papers also examined behaviours [71,72,73], perceptions of products [71,73,74], and preferences [72] of customers during their VR grocery shopping. The subject of VR stores was also discussed in the literature from the perspective of designing such shops and services. The paper of Sun et al. [3] explored the design of VR grocery stores to meet individual needs, using a co-design workshop where participants brainstormed aspects like product displays, navigation, shopping carts, and social shopping. The authors discussed two main design directions for VR grocery shopping: simulating existing physical stores and creating new virtual stores. According to Violante [75], the VR environment presenting a virtual supermarket in the form of 360-degree video can create highly immersive sensory experiences that promote the presence of consumers and impress their senses, touch their hearts, and stimulate their minds.

On the other hand, there are and will also be many limitations connected with VR technology and this type of shopping. First of all, there is the issue of accessibility. Such solutions require access to VR hardware and technology, which may not be widespread. Then, high-quality VR experiences require significant computing power and fast internet connections. Another problem is related to social adaptation and barriers. Users may need time to adapt to using VR for shopping. Moreover, not everyone can use VR technology due to health contraindications. Currently, Virtual Reality is not a suitable technical mean for everyone. For example, people with epilepsy or motion sickness cannot put on goggles to use them. Of course, as technology develops, some problems will be solved, but new ones will also appear, for example, related to legal or ethical issues.

Reviewing online shopping solutions using Virtual Reality, the concept of the metaverse should also be mentioned. The metaverse, a collective virtual shared space created by the convergence of virtually enhanced physical reality and physically persistent virtual spaces [76,77], has transformative potential for online shopping. Within this immersive digital realm, users can engage in interactive and realistic shopping experiences that blend the convenience of online commerce with the sensory and social aspects of physical shopping. Shoppers (already) are and (probably) will be able to:

- navigate through virtual stores using avatars;
- interact with 3D models of grocery products;
- receive real-time assistance from virtual sales representatives;
- experience interactive product demos;
- access personalized recommendations;
- benefit from advanced search options;
- create their own shopping list;
- interact with other people in the virtual shops—for example, their family, friends etc.

**Figure 5:**
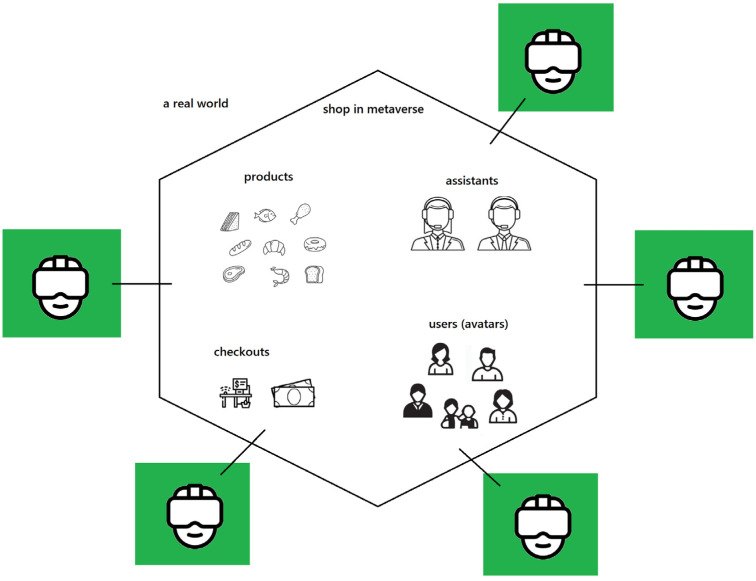
The concept of shop in metaverse [own work].

Customers in a virtual store in the metaverse using Virtual Reality technology are in a different reality, feeling like they are in a store. The metaverse store can virtually reproduce a real store. Customers can move between shelves, look at products, meet other people, get some help from virtual assistants, and have a preview of their product cart. Figure 5 shows the concept of shopping in the metaverse.

This integration not only enhances user engagement but also offers personalized shopping experiences through advanced AI algorithms and data analytics. Additionally, the metaverse facilitates social shopping, allowing friends and family to shop together in a virtual environment regardless of physical distance. As technology advances, the metaverse can revolutionize the retail industry, providing a shopping experience that bridges the gap between the physical and digital worlds.

There is already research in the literature that examines the behavior, will, and motivation of shopping in the metaverse, for example [78,79]. There is also research on scenarios aimed at reducing social isolation among older adults, highlighting the importance of considering social interactions in metaverse modeling [80]. Even customers’ e-satisfaction towards grocery shopping in the metaverse has been already studied [81]. This shows that Virtual Reality and the metaverse in the context of shopping for groceries is not just a vision of the future but is already a practice, and some companies already offer such services.

However, there are certain barriers to using VR and the metaverse, including:

- high hardware costs that limit accessibility to a wider consumer base;
- the need for powerful hardware and fast internet connections that may not be available to all users;
- the lack of VR and metaverse skills, as well as technical issues with using them (registration, login, applications, passwords, navigating the platform, problems with understanding the logic of the virtual world);
- challenges related to social adaptation (technological advances, fear, resistance, skepticism, distrust, etc.);
- health contraindications to be in VR/the metaverse (e.g., motion sickness, epilepsy, pacemaker, and others), as well as psychophysical effects related to the use of hardware (e.g., headaches, nausea, dizziness, fatigue, eye pain, and many others);
- the potential need for training (guidance) for users to navigate VR environments;
- users’ reluctance to collect data on their consumer behavior, to monitor and control them, etc.;
- limited interactivity of products, which may limit their attractiveness;
- from the perspective of creating stores: significant investments in software development, server updates, and maintenance.

Finally, there are ethical issues to consider when it comes to grocery shopping in Virtual Reality and the metaverse. These include a range of issues, especially those related to data privacy, monitoring, and control. These platforms and applications collect vast amounts of user data, including shopping preferences and behavioral patterns, as well as product and service ratings, raising concerns about how this data is used, stored, and shared. There is also the issue of Artificial Intelligence. Such solutions can be AI-based, which opens up a range of risks related to fake news, customer manipulation, and subliminal messages. Just using websites creates many opportunities for cyber threats, and a fully virtual, closed world in which a person can “live” as an avatar is even more filled with such issues.

### 3.4. Smart Devices and AI in Food Ordering

Application of high-technology functions. Many of these devices are driven by Artificial Intelligence and machine learning algorithms, designed to make ordering groceries or even meals from restaurants easier [82]. As more and more homes become integrated with “smart” technology, from voice assistants through touchscreens on refrigerators to automated delivery systems, the way in which people can approach food shopping and dining is rapidly changing [83]. Table 1 shows a characteristic of the main food ordering devices.

One of the most significant advantages of food ordering smart devices is the level of convenience that they offer. With a simple voice command or touch on a screen, users can get different options—from basic groceries to exquisite meals from local or national restaurants [84]. Devices like Amazon Echo, Google Nest, or smart refrigerators allow users to not only order food but also to manage their household inventory by tracking expiration dates, suggesting recipes (for example, based on available ingredients), and even offering nutrition advice [85]. That creates a more user-friendly experience where many tasks are taken care of with the minimum effort possible [86].

These devices are increasingly integrated with online delivery platforms, which offer very easy access to these food services. Smart devices, using sophisticated algorithms, can learn from past behaviors and preferences to make recommendations for products or meals that better fit the taste of an individual. This level of personalization not only enhances the user experience but also reduces decision problems because people no longer need to scroll over long lists of products or menus [87,88].

Food-ordering smart devices also change how households manage their budgets and consumption habits. Many devices have budget-tracking features. They allow users to set limits on money spending or compare prices in different stores and restaurants [89,90]. This helps users make more informed decisions and avoid impulsive purchases. These can lead to reduced food waste. These devices can also contribute to sustainable practices through the suggestion of environment-friendly products or through the promotion of local food that fosters a more responsible behavior in food consumption [91].

Other major benefits are the time that is saved from automating this process. Busy households—especially families or people with hard jobs—can reorder groceries or routine meals without having to take the time to search for each item manually. These will link accounts from multiple delivery services or grocery providers to ensure users can get their food in a timely and efficient manner. It comes with automated reminders for reordering or subscriptions to commonly used items to reduce supply monitoring to a minimum, giving users time to rest and not worry about it [92,93].

As the technology evolves, the future will probably offer more possibilities for food ordering from homes [94]. Immersive technologies like augmented reality [79,95,96] and Virtual Reality [3,79,97,98] could change a lot the ways users interact with their food, allowing them to virtually explore restaurants or grocery stores and then make a purchase. In addition, advanced AI will enable these devices to perform tasks that better satisfy needs in diets or suggest healthy options, thus making food choices aligned with users’ objectives of wellness.

There are also many considerations that have to do with ordering devices for food. First, there are privacy issues, including the collection of data. These devices often track personal information such as shopping habits and diet preferences. It is important for users to be aware of how their data is used and if the appropriate safeguards are used [99,100,101,102]. Furthermore, the use of these devices may unconsciously undermine some of the traditional, practical aspects of cooking and food preparation, which may have a negative impact on culinary skills and the social value of communal meals.

**Table 1:** Characteristics of the main food-ordering devices.

Device	References	Characteristic
Smart refrigerator	[103,104]	They are modern refrigerators with touchscreens, cameras, and sensors that monitor food stock and expiration dates. These devices can suggest recipes based on what ingredients are available and notify users when it is time to restock certain items. They also often are synchronized with delivery applications, making reordering easy. Some models have voice control and Smart Home integration, allowing users to order food using voice assistants or even see the contents remotely via smartphone apps.
Voice assistants (e.g., Alexa, Google Nest)	[104,105,106,107,108]	They are voice-controlled gadgets that enable touch food purchasing, allowing users to easily order groceries or meals from eateries by speaking commands. These devices connect with delivery services and keep track of past orders while suggesting personalized meal or grocery options based on previous choices. Additionally, they can remind users to reorder items and help create shopping lists as well as suggest healthier alternatives based on diet preferences.
Smart ovens	[109,110,111]	Some ovens have sensors and AI, offering automatic adjusting of cooking temperatures and times based on the dish prepared. Some models integrate with food delivery and recipe apps, allowing users to download and follow cooking instructions directly from the oven’s display. These ovens can even suggest recipes based on the user’s dietary preferences and help them order missing ingredients directly from partner grocery services. They often come with remote control options via smartphone apps.
AI-powered meal planners	[112,113,114,115]	They offer advanced algorithms to analyze dietary preferences, restrictions, and past meal choices to recommend personalized meals. These planners can be linked to grocery delivery services, making it easy to order all the ingredients needed for the selected recipes. Some systems even track the user’s health metrics, such as calorie intake and nutrition goals, to ensure the meal suggestions align with fitness or health objectives. The planners learn over time, improving recommendations based on evolving tastes or habits.
Smart speakers with food apps	[116,117,118,119,120,121,122,123,124]	They are advanced meal planning tools that analyze diet preferences, restrictions, and past meal choices to offer personalized meal recommendations. Many of these tools can connect directly to grocery delivery services, simplifying the process of ordering ingredients for the selected recipes. Some systems go a step further by tracking health metrics like calorie intake and nutritional goals, ensuring the suggested meals support fitness or health objectives. Over time, the planners adapt, refining their recommendations based on changing tastes or habits.
Smart displays (e.g., Echo Show, Google Nest Hub)	[125,126,127]	They are equipped with both visual and voice interfaces. These devices allow users to not only order food but also visually search restaurant menus, follow recipe tutorials, and check their grocery list. They offer video call functionality, which can be used, for example, for virtual cooking consultations or connecting with chefs. Users can watch recipe videos while cooking and receive real-time guidance, making meal preparation more interactive and engaging. Smart displays are often synchronized with other smart kitchen devices to get complete integration.
Automated grocery scanners	[128]	Devices, whether stand-alone or built into smart refrigerators, scan grocery barcodes as items are added or removed from the fridge or pantry. This system tracks the inventory and updates a digital shopping list or automatically reorders items when they start to run low. Some devices also suggest alternatives based on availability, cost, and user preferences, with options to prioritize local or eco-friendly products.
Subscription-based smart devices	[129]	These are services that automate the reordering of frequently used items through subscription services. Users set preferences for how often they need specific items (e.g., coffee, cleaning supplies, snacks), and the device ensures timely delivery without the need for manual reordering. These devices often sync with Smart Home systems, providing notifications when subscriptions are due for delivery or offering options to skip or delay shipments, based on current inventory. They are ideal for households with predictable consumption patterns.
Smart food scales	[129,130]	Modern scales that connect to food-preparation and recipe apps help you accurately measure ingredients. They can assess portion sizes, give nutritional details, and suggest recipes based on the ingredients being weighed. If something is missing, the scale can add it to a shopping list or even place an order with a connected grocery service. Some smart scales also support dietary goals, offering portion-control tips or healthier ingredient alternatives.
Smart delivery lockers	[131,132,133,134]	These are secure lockers that are temperature controlled. They are installed outside homes, and their role is to receive food deliveries when no one is available (for example, no one is at home or in the office) to take them. Thanks to these lockers, groceries or meal deliveries remain fresh and safe, preventing issues like spoilage or theft. They are especially useful for easily spoilt goods and can be linked to a user’s food-ordering system. They can automatically update the status of orders and notify users when a delivery is completed. Some models also offer remote access for couriers.

Source: Authors’ own work on the basis of: [103,104,105,106,107,108,109,110,111,112,113,114,115,116,117,118,119,120,121,122,123,124,125,126,127,128,129,130,131,132,133,134].

When it comes to the most innovative developments in the area of smart food ordering home appliances, the smart refrigerator must be mentioned. This device has changed a lot and evolved much beyond its traditional role of food preservation [135]. This modern version can be observed as a sophisticated food-ordering appliance, integrating convenience and technology to enhance the user experience. The main features of smart refrigerators are presented in Table 2.

At the top of the smart refrigerator’s functionality, there is its ability to monitor and manage food inventory [136]. Equipped with advanced sensors and cameras, the smart refrigerator follows the items within its compartments in real time by constantly updating and providing an overview of its contents to the user. This reduces wastage of food through suggestions on what items are approaching their expiration date for use. It makes meal planning easier since it provides suggestions based on the available ingredients [137,138].

A very important fact is that smart refrigerators are integrated with online grocery shopping platforms. Using the internet, this device can facilitate the ordering of groceries directly from the refrigerator’s interface [139]. Smart refrigerators are increasingly offering integrated AI capabilities in the improvement of food-ordering capabilities. They are also capable of analyzing usage and consumption patterns, projecting future demands through the presentation of recommendations, or by automatically generating grocery lists. For example, if it detects that certain products, like milk and eggs, are almost expired, it may recommend such products for purchase and perhaps even place the order [140]. Also, smart refrigerators are frequently integrated with AI features that enhance their food-ordering capabilities. These systems can also analyze usage patterns and consumption habits to predict future needs through the offering of recommendations and the automatic generation of grocery lists. For instance, if it detects that products like milk and eggs are running low in the refrigerator, it can suggest buying them and may even make the order itself [82,83].

Voice-controlled assistants in smart refrigerators enhance their ease of use [84]. Users can address commands to it, for instance, to check the inventory, give some ideas about recipes, or just let the fridge order some products. This feature makes it easier for users to operate this appliance and therefore manage tasks in a more efficient way [85,86].

Smart refrigerators can bring an impact on having a more organized and effective kitchen space. Through this digital interface, one can get access to the following features: recipe databases, meal-planning tools, nutritional information, etc. In this way, they provide support for making rational food choices, including healthier eating. This could be beneficial in supplying detailed information on the nutritional content of products [87].

**Table 2:** The main features of smart refrigerators.

Feature	References	Characteristics
Real-time inventory monitoring	[82,141,142,143]	Smart refrigerators are designed with sensors and cameras that give a constant overview of the content: the products inside. Further, this technology allows users to remotely perceive what is in their refrigerator through either a smartphone app or the appliance’s interface. Due to this, users will know their level of stock without having to check it by hand. This works particularly well to manage groceries and ensures that one does not end up overbuying or running out of items.
Automatic expiration alerts	[85,86,87,88]	These appliances will be able to detect when some particular food items are near their expiration dates. If products are about to expire, the system will automatically notify the user. This function enables smart refrigerators to reduce food wastage by using items in time. This feature also enables the setup of custom notifications or reminders based on the user’s preference.
Online grocery ordering	[84]	Smart refrigerators can be integrated with online grocery platforms. They let users view, select, and purchase groceries from a UI or application. By doing so, making a list and going to a shop physically would no longer be necessary. It thus makes it way easier to keep up a kitchen. The user often is able to select from his or her grocery stores of preference. They even schedule deliveries.
Personalized recommendations	[83]	Smart refrigerators come equipped with AI and analyze user preferences, dietary restrictions, and current inventory status to make personalized meal and shopping suggestions. The devices support the exploitation of the best ingredients already on hand and take some of the work out of meal planning. This is a very helpful feature for people who like to try new recipes or make their grocery shopping much more efficient.
Voice control integration	[140,144]	Many smart fridges come equipped with widely used voice assistants, like Amazon Alexa or Google Assistant; this way, users can interact with the device using voice commands without touching it. The user can query the refrigerator to find out what is in its storage that is running low, add it to the grocery list, or research a recipe.
Artificial Intelligence	[100,101,102]	Artificial Intelligence in smart refrigerators works out the user’s behavior and consumption patterns to predict their future needs. It analyses past purchases and trends of use, and it may suggest shopping lists and can even automate orders for items that are in high demand. In this way, users will never be caught off guard and will avoid the risk of running out of items.
Recipe database access	[135,139,145]	Most of the intelligent refrigerators can already access great databases for recipes. With these, you could look up specific recipes that contain ingredients that you have in stock, that are fit for a diet, or that meet particular nutritional requirements. All such features make meal planning more efficient and might even encourage healthier eating—the detailed recipe and the ingredients list come right to you. Other models may include step-by-step cooking tips and offer video tutorials.
Nutritional information display	[139,145]	These can show the complete nutrient information about the products stored, ranging from calorie counts to macronutrient amounts and other nutritional measures. This could be helpful in making diet-related rational choices to maintain a perfect balance in the user’s diet. It could enable the easy display of this nutritional information on the refrigerator or through some special application.
Digital interface	[101,102,143,146]	The digital interface for a smart refrigerator is normally manifested in the form of a touchscreen or an application interface. It allows the user to interact and manipulate the various features provided by the appliance. This web page makes navigation effortless for inventory checks, setting preferences, and other functions, including integration with calendars, shopping lists, and even recipe management.
Smart Home integration	[142,143,146]	Smart refrigerators may be integrated with other Smart Home devices. This, therefore, helps in bringing into place complicated and connected home environments. They may be integrated with smart lighting, thermostats, and security cameras.

Source: Authors’ own work on the basis of: [82,83,84,85,86,87,88,100,101,102,135,136,137,138,139,140,141,142,143,144,145,146].

Smart refrigerators with food-ordering features are becoming increasingly popular. It is worth noting that they can include features and applications tailored to regional markets. These devices are an example of integrating modern technology into everyday kitchen management. Such solutions increase user convenience. For example, in the United States, it could be Samsung’s Family Hub (Suwon, Republic of Korea), integrated with an intelligent refrigerator that enables the owner to place a food order on it [147]. This model comes with an integrated touchscreen that makes it easy to perform other tasks—for instance, browsing recipes, creating shopping lists, and ordering groceries. Through partnerships with major food retailers like Amazon Fresh and Walmart, users can manage their inventory and place orders directly from the device. This integration provides real-time updates on inventory levels and expiration dates [148].

Another example comes from South Korea: It is LG’s InstaView smart refrigerator with its SmartThinQ application. The refrigerator has a glass display for the user to interact with the appliance through it. This easily lets the user see what’s inside without having to open the door [149]. The SmartThinQ application allows users to check inventory, receive notifications of expired items, and order groceries online from various delivery services [150]. Another example of smart refrigeration, with the possibility of ordering food, is Germany’s Bosch Home Connect. The Home Connect application enables users to monitor the contents of the refrigerator, notifies them about items that are approaching expiration, and even places orders for groceries from local stores. The technology is integrated with grocery services, providing users with a way to manage their food [151].

Panasonic’s smart refrigerator, including the concept of Internet of Things, offers real-time food monitoring in Japan. One can download a particular smartphone application, which also provides automatic updates about expiration dates and makes online grocery orders comparatively easy. This helps integrate with local retailers and delivery services to enhance the device’s functionality [152,153].

For Europe, there is the Miele Smart Fridge from Germany; it works with the Miele@home application, whereby users can keep track of inventory, receive notifications of expiration dates, and know where to purchase groceries online through partnered services. In this direction, Miele enhances user convenience by means of today’s modern technology [154,155].

Table 3 presents the different applications of smart refrigerators in different countries. It also highlights their roles in changing food management and ordering practices.

The appearance of smart refrigerators as food-ordering devices is a big step forward when it comes to household convenience [88] (Table 4). These devices offer a range of benefits that are fundamentally changing the way people shop for food and groceries. They make these tasks easier and more efficient. A major benefit with smart refrigerators is convenience for the users: integrated directly with online grocery shopping platforms, users can browse, select, and order groceries right from the kitchen without having to leave [100]. There is no need to manually create lists or constantly make trips to the store. Accordingly, by reducing the time and effort in managing home supplies, a few touches on the touchscreen or a voice command helps the user replenish supplies more efficiently [101].

Another key benefit associated with smart refrigerators is time savings. Thanks to automated food tracking and ordering, users spend less time shopping for groceries [102]. Features like real-time food monitoring and automatic ordering of products make shopping more comfortable. This is especially beneficial for busy people. Additionally, smart refrigerators help reduce food waste by sending messages (alerts) about expiration dates of products. Also, they provide real-time inventory updates [144]. A proactive approach ensures that food is used before it spoils, resulting in less waste and a more cost-effective kitchen [141].

Other important features enabled by smart fridges include personal shopping. By applying AI, the devices analyze the user’s preferences and consumption patterns, even dietary needs, for better shopping recommendations [141]. This personalization results in users receiving suggestions and orders that match their individual preferences, making grocery shopping more relevant and aligned with customers’ needs. Helping with meal planning is another advantage. Thanks to access to online recipe databases and real-time inventory information, users can easily plan meals. This can be done based on what they already have in their fridge. By recommending recipes that use available ingredients, smart refrigerators contribute to a more organized and well-planned kitchen environment [142,145].

Smart refrigerators inform users when items are running out. Thanks to this, they avoid situations in which they run out of some products [136,143]. That would mean that ingredients are always available inside the refrigerator, thereby minimizing problems during meal preparation because of a lack of supplies. Smart fridges also help reduce shopping errors. The automatic ordering of frequently used items, based on historical data, minimizes the chances of forgetting groceries. In this case, automation ensures that at all times, users have the required supplies, thereby avoiding that last-minute trip to the store [86,87].

Another advantage is that it is more accessible. Voice control and intuitive digital interfaces enable users to approach their refrigerator in a more interactive and user-friendly way. Besides, the smart refrigerator contributes to managing shopping more easily. This could be particularly useful for people who have mobility difficulties or who prefer a more modern kitchen [146,156]. Smart refrigerators may support healthy food choices by informing users continuously about nutritional information and producing personalized suggestions related to cooking and recipes. Such features enable users to make better choices regarding their food intake. This can also support their health goals and dietary needs [136,140].

The emergence of voice assistants from the likes of Amazon Alexa and Google Nest has really revolutionized people’s interaction with technology (Table 5). AI indeed has influenced the element of lifestyle very well. More often than not, smart devices have an easy-to-use interface that in turn interacts with a variety of services. Modern ways of ordering food that are possible today were not previously possible using traditional methods [157,158,159]. Voice assistants are designed to process natural language. Voice assistant technology allows users to communicate with their devices as they would with another person. As such, ordering food, for example, becomes very intuitive and painless, as the user can order with his voice anything from their favorite restaurants or food delivery services without having to navigate through complicated menus or interfaces [160].

Voice assistants can provide updates of a different nature. For example, they can be real-time status updates on orders, such as estimated times of delivery. What is more, they may update the customer on the status of the order without having to constantly use smartphones or any other devices. Such assistants are often integrated with various food delivery apps [161]. Further, the ability to place orders by voice assistant extends to grocery shopping to add an item in a shopping list. Thus, voice technology is versatile and adaptable to a variety of uses concerning food procurement and consumption aspects, accordingly [162].

Presently, Amazon Alexa is one of the most popular voice assistants in the United States, while it also has been used for food ordering. The major food delivery services include Domino’s Pizza and Grubhub, integrated with this assistant [163]. For example, a user can say, “Alexa, order a large pepperoni pizza from Domino’s”, and Alexa will do it. First, it will access Domino’s application, then manage the order, and finally handle payment. Users can be informed about the order in real time. The device also uses historical data to suggest reorders or new menu items based on previous preferences [164,165]. Of course, Alexa helps operate a variety of other home devices. While waiting for their meal, users can adjust the lights, control the thermostat, or set cooking timers—all via voice commands [166,167,168].

Another example is, of course, Google Assistant. Just like food ordering in the UK, Google Nest—which comes with Google Assistant—can do the same. The device integrates with popular platforms like Deliveroo and Just Eat, so users will have the ability to issue commands to the assistant by using phrases. Examples include: “Hey Google, order sushi from Deliveroo” or “Hey Google, find me a pizza on Just Eat”. Google Assistant also has access to the list of previous orders and the user preferences, and hence, it can provide recommendations. It also places an order, pays for it, and tells the user about the status of the delivery, just like Alexa. This assistant also helps in areas of a person’s life other than shopping [168].

The third most popular example is, naturally, Siri. It is mostly in the United States (Apple HomePod), but this technology is now spread and people around the world use it. Users can activate Siri, for example, by saying “Hey Siri, order dinner from Menulog” or “Hey Siri, get me a burger from the nearest restaurant on Menulog”. Siri will proceed with the ordering through the Menulog application and will even process the payment. Access to data enables Siri to suggest restaurants and menu items to the user. It can, hence, be said that voice assistants enhance the personalization of the food-ordering process [114,115,116]. Siri integrated with other HomeKit devices from Apple lets users control the environment within their homes while controlling their food orders. This integration of the ordering of food with home automation shows the various facets associated with the application of this technology in routine everyday life [169,170].

The use of smart devices and AI in food ordering brings about convenience, automation, and personalization in grocery shopping, but at the same time it raises several data privacy concerns related to user acceptance [171,172]. Most of these devices collect a wide array of sensitive data that includes consumption patterns, dietary preferences, health information, and inventory levels within a user’s household [173]. While this will provide personalized recommendations and automate reordering, it also raises a number of questions around data security, user control, and privacy risks [174,175]. These need to be weighed against the potential benefits if users are going to trust and accept such technologies (Table 6).

Important challenges arise in terms of data integration and safety that arise from such smart devices. The kind of data retrieved is personal; therefore, effective measures of securing them, such as encryption and storage in secure clouds, are urgent needs to guarantee users’ privacy [122,181]. Security breaches lead not only to the leakage of user data but also to lost confidence in these technologies. Therefore, it requires companies to shift their focus toward providing better practices of secure data storage and providing users with a clear policy of how their data is managed in order to let them feel assured about their security. Once users understand precisely how their data is being stored and secured, they would be much more accepting of having these smart devices in their homes [182].

Other critical drivers of adoption relate to how users can have control over the collection and diffusion of data. If users perceive that they lack control or understanding regarding how their data are used, then they might resist the adoption of such devices. Providing choices for users in terms of privacy settings and options with regard to management of the data collected allows them to feel in control of their personal information [176]. Moreover, transparency into how AI-driven personalization works can help consumers understand and appreciate the benefits it may generate. For example, a simple explanation of how data is used to either personalize recommendations or reduce food waste can turn an AI-driven process into an even more helpful but less invasive one [183,184].

Another transparency factor in the use of data involves user acceptance, especially on the matter of third-party access. Most smart devices for ordering food will interact with third-party providers like grocery delivery services [185,186]. Without a clear explanation of how data is shared, it would contribute to extra privacy risks [176,177]. Users are also likely to tolerate simpler privacy-breaching practices if the terms are understandable and they may reap benefits that could be tangible either by saving money or time. Well-set policies in place with regard to third-party access, and the ability for opting-in in relation to sharing of data, would help users make better choices while generally feeling comfortable with the use of the technology [178].

AI personalization taken as an entity is something that really needs to be given some careful thought. Though AI personalization enables recommendations that can be much more relevant, along with timely reordering, if users are not adequately informed about how their data informs these processes, there lingers a feeling of intrusion into privacy. If users feel that the recommendations by AI are too targeted, they may resist the use of the devices due to anxieties over privacy concerns. In this regard, firms should make it crystal clear how AI will improve the shopping experience. Generally, showing users through demonstrations how AI personalization positively affects them—whether through reduced food waste or optimization of inventory—enhances understanding and reception [176,179,182].

Another likely evidence of confidence for users would be data-protection compliances, such as the General Data Protection Regulation. Conformity to this kind of regulatory standard also helps assure users that companies will treat the rights of users with consideration and handle data with responsibility [175,187]. Clearly, data-retention policies can better raise the comfort of users, as indefinite storage of data may give rise to situations that are unwanted, where data may be misused in some way over a long period. Reasonable periods for retaining data or allowing the deletion of data after a particular period show regard for user privacy that can increase user acceptance. Data privacy and security concerns, therefore, form the bedrock for the wide acceptance of smart devices and AI in food ordering. When these in-store technologies offer clear benefits to the users, and the latter understand how their data is protected and used, they are likely to more warmly embrace those in-store technologies. Transparency about data practice, giving the user control of settings, and compliance with privacy regulations provide the base for trust and acceptance enablement, especially for smart devices and AI to unfold their potential in grocery [172,173,175].

## 4. Discussion

From manual to technology-based grocery shopping, the turn is huge in consumer behavior and industrial approaches. This review further elaborates on how the amalgamation of emerging technologies—Artificial Intelligence, Virtual Reality, and voice assistants—has rearranged not only how consumers interact with stores but also their expectations for convenience, personalization, and immediacy.

It is in this respect that one of the strongest themes that emerges from this study is related to the increasing orientation towards consumer convenience. The study of the literature on the subject has revealed and, further from the analysis of various supermarkets and their online platforms, it is crystal clear that this is an area that has grown in a quite phenomenal manner because it can give every consumer a more personalized, even efficient, shopping experience. From in-store experiences chained to self-service checkouts and handheld scanners, through mobile applications, augmented reality, and AI-powered recommendations, the fire has been lit. These move toward hyper-personalized shopping environments where customer preferences and habits truly drive the retail experience, driving frictionless exchange between the consumer and the retailer.

But with Virtual Reality and the metaverse in the mix, that goes into a completely new dimension, even beyond convenience. With immersive virtual grocery stores, this can just get the world of shoppers through a completely different experience. Companies like Tesco have proved, along with other international retailers, that this VR solution will enable users to walk down virtual aisles, inspect products, and even communicate with virtual sales assistants. Grocery shopping in the metaverse is at that cusp, finally reaching the place that would showcase the best elements of human, hands-on retail combined with the efficiencies and conveniences of online platforms. Not all kinks have been ironed out just yet, especially with regard to aspects of access and technical infrastructure in support of good VR experiences. It is because of resource-intensive computing power, fast internet connections, and VR hardware that these solutions might become available to no one other than an increasingly technologically endowed population. This is further underlined by the spread of food-ordering smart devices, such as refrigerators with AI and the ability for automation and Smart Home ecosystems [188,189]. These devices make it convenient not only to order groceries but also to fit into the user’s daily routine: monitoring inventory, suggesting recipes, and automatically placing orders. Sure, these devices are convenient; but considering them in a wider perspective raises very valid questions regarding data privacy and security. While this equipment records large volumes of personal data, such as consumption patterns and shopping habits, there should be an explanation of how that data is used and stored in view of guarding users’ privacy.

Farm-to-table and Blockchain-based marketplaces also remain very relevant. With consumers becoming increasingly conscious of sustainability and locally sourced products, technologies supporting transparent and direct interactions between farmers and consumers become more relevant. Concretely, Blockchain would be able to guarantee traceability and authenticity, which would allow for better consumer choices, consequently reducing food waste. However, for these and several benefits to come into full realization, there is a need for such innovations to take into consideration issues to do with access to the internet and technological literacy on the part of the farmers themselves.

With that said, what this review has made clear in all aspects is that a nexus of technologies purposed to make the consumer more convenient and engaging will characterize grocery shopping in the future. This shall increasingly be so with the inclusion of AI, VR, and Smart Home devices in shopping, where this will not be a chore but integrated in daily life because of automation and personalization. While these developments are very promising, the discussion of inclusion and access, together with concerns about data privacy, will continue to feature in the future of grocery shopping. Future research should be oriented toward how to make these technologies more accessible and safer for all consumers to benefit from such innovations.

The changes which have taken place with Virtual Reality shopping due to online purchases is that this kind of shopping has turned both immersive and interactive, in the manner in which consumers come into contact either with products or with the shopping environment [187]. Whereas traditional e-commerce is characterized by scrolling on products listed on a website or a mobile application, VR techniques create a three-dimensional virtual simulated environment where customers can virtually enter the store for the purposes of exploration, interaction with goods, and coming closer to in-store shopping without leaving their homes [190,191]. This basic difference in conception thus illustrates a number of profound differences in terms of relative advantages and challenges facing VR shopping in comparison with traditional online shopping [3].

Other differences between VR and classical online shopping are the interactivity degree of the product and the degree of involvement. The customer would browse through virtual aisles and see items in a realistic, three-dimensional format [70,192]. Products can be rotated and viewed from all sides. In this interaction, it will be possible for users to create a sense of physical presence in the simulated store experience in VR, while at the same time improving their connection with the products under consideration. For instance, in a VR online grocery store, one can “walk” down virtual aisles, pick up products and read labels close up, and place items into a virtual shopping cart [193,194]. Traditional online shopping, on the other hand, restricts consumers to a two-dimensional interface in which images and descriptions of products are viewed—a thing far less engaging than the ability to examine an item in person [195].

Besides, Virtual Reality shopping can be much more flexible and customizable into highly personalized experiences that best fit the taste and behavior of an individual. For example, in VR, retailers will be able to offer personalized store layouts or even suggestions for certain products by considering preferences shown by a user in previous virtual shopping experiences [196,197]. That would mean immersive personalization could go one level further than just recommendation algorithms added to traditional online shopping with experience to improve customer satisfaction and engagement. Also, VR shopping will be able to include social features where people shop with friends or with virtual sales assistants [187]. This creates a particular shopping experience, one that relates more to social aspects of in-store shopping than to the usual loneliness of online shopping [198].

Of course, there are also quite a number of significant barriers to the widescale adoption of VR shopping—issues that do not burden traditional online shopping. There are some very real limitations brought about by dependency on equipment: VR depends on headsets, controllers, high-speed internet, and a compatible device able to run the virtual environment with ease [3,199]. This is actually one of the downsides for consumers who may not have all this technology available at their fingertips and who find traditional online shopping so much easier. Traditional online shopping requires no more than a basic level of access to the internet and some device, such as a smartphone or a computer, which makes it so much more accessible. Last but not least, VR shopping can sometimes cause motion sickness or discomfort, and hence, limits its appeal for some groups of users, while traditional online shopping does not create any kind of physical discomfort for the user [200,201].

On the convenience side, traditional e-commerce remains an easier and faster way to buy something arguably because no setup or special devices are required. For many, it feels seamless and fast to navigate through websites or apps, add items to a shopping cart, and confirm a transaction [3,202]. As VR shopping entails deeper, more immersive engagement that can indeed be enriching, it is not good when there is a need for quick or repetitive shopping. While consumers currently may find the VR equipment prohibitively expensive to justify frequent grocery shopping or other frequent purchases with the technology, another key difference between VR and online traditional shopping has to do with consumer behavior as it pertains to making purchase decisions. Immersive VR can make people feel more attached to the products. It will translate into higher confidence in purchases [70,203]. The ability to view merchandise “in person” when visiting virtual stores can eliminate much of the hesitation of some consumers to make online purchases, especially items where size, appearance, or quality is an important consideration. Traditional e-commerce, while efficient, relies strongly on consumer reviews and detailed product descriptions and photos for establishing trust, which may be partial in replacing the confidence that consumers get from a VR experience [3,191,192].

Table 7 present a comparison between Virtual Reality (VR) shopping experiences and traditional online shopping.

The effectiveness of online grocery shopping technologies is based on their ability to significantly enhance both the convenience and personalization of the shopping experience. That would be efficient if, with the advancement of technologies meant for online grocery shopping, the ease and personalization of the experience increased manifold—at least tenfold. Artificial Intelligence changed dimensions in the sphere of consumer-to-grocer communication [204]. AI-driven online platforms push recommended personalized messages, predictive analytics-personalized shopping experiences in tune with behaviour and preference of the consumer. This therefore creates an intuitive purchase area where consumers can always find something that suits them without necessarily having to look for them. Practical examples could include AI-driven inventory tracking and automatic re-orders. Those systems will save the time and effort of the consumer in ensuring they never run out of the basics [205,206].

It is here that online grocery shopping technologies have caught up with the rising demand of consumers for immediacy and flexibility. Examples include smart devices, ranging from refrigerators monitoring inventory levels down to where they send notifications when they perceive reorder levels [99]. Added to this are grocery platforms that allow the shopper an easier and quicker way of shopping, whereby one attends to other businesses in life while groceries needs are taken care of. Adding to this level of convenience are voice assistants, which allow for hands-free shopping where the consumer multitasks as he or she places orders [207].

These are going to attend the rise in demand for personalized experiences. The accruing benefits from these technologies, therefore, are not going to stop at mere convenience. For example, AI will analyze big pools of data and make very realistic predictions about consumer preferences and purchasing habits. It can even go ahead and suggest some products for achieving satisfaction with an individual taste, dietary restriction, or health goal [208,209,210]. Equally, VR and the metaverse will be monetized in making shopping experiences more immersive. These will connect the virtual and physical worlds of shoppers through their browsing of groceries in a more entertaining and interactive style. New technological developments now make it possible for customers to shop in the way that best meets their expectations of seamless personal experiences [3,192,199].

Of course, the general acceptance of technologies for online grocery shopping brings forth several disadvantages. While millions of consumers are embracing digital tools, part of the population is devoid of the required technological wherewithal, access to the internet, or even financial capability for online navigation. Barriers could also signify the collection of obstacles that culminate in specific demographic exclusion from online grocery shopping. Hence, there are differences between those who have advanced technologies and those that do not [192,193,204].

Other major concerns of the digital grocery shopping arena are those that deal with privacy and data security. It would involve vast volumes of personal information, from buying habits to food preferences, and even health data [16]. This fact has raised a number of questions as to how this information would be stored, disseminated, and kept safe. Misuse of data related to sensitive information, in light of the increasing number of reports on data breaches, will make consumers vary of trusting the retailer with recording their personal information. It will challenge the retailer to protect the data, consequently, and hence, make it clear to the customer what happens with their data [3,179,180,187,199].

While this might be much more convenient to shop for groceries online, this could add up to more environmental burdens: larger delivery services—let alone smaller but more frequent orders applied to higher numbers—raise the total carbon footprint resulting from packaging and transportation. This will definitely no longer be sustainable if some of the stores try to adapt to eco-friendly packaging and carbon-neutral delivery options. This is one variety of convenience at an environmental cost; the trade-offs between convenience for such environmental costs have to be weighed judiciously as further development takes place in this sector [204,205].

The other weakness may be technological limitations: though AI and VR indeed are rather well-developed, they nonetheless are far from perfection. For example, some recommendation systems based on AI sometimes give recommendations that are wrong, or they cannot include complex user preferences. Besides, shopping in VR might turn out not so convenient or may not appeal to every consumer unaccustomed to virtual surroundings or without the appropriate hardware at their disposal. For example, VR headsets are part of this hardware [3,187,190,191,198,199,200,201,211].

Table 8 summarizes the analyses about the advantages and disadvantages of the main online grocery shopping technologies.

The potential for online shopping technology innovations is massive since everything goes in tune with the needs and expectations of digital-savvy customers. Online shopping becomes personalized, productive, and even more engaging with the use of mobile technologies like AI, VR, voice assistants, and smart devices, just to mention a few, thereby permanently altering consumer behavior in relation to retailers [3,187,191,192,211]. All of these technologies promise a smoother and more pleasant shopping journey for users, hence leading to greater convenience and satisfaction for them. Artificial Intelligence is important in making online shopping more intelligent. It really allows different platforms to understand and predict consumer preference [198,199,200,211]. Advanced algorithms, based on a great volume of data analysis, recommend products to an individual based on his or her previous purchases, browsing history, and even personal preferences. This creates an extremely customized shopping experience in which the consumer is exposed only to what would be relevant for them, thus minimizing the time they must spend to search for products and, therefore, making the whole act of shopping much more efficient. Besides that, AI-powered personalization empowers retailers to show dynamic pricing and promotion—a guarantee that every customer gets the most relevant deal and offer regarding their shopping habits [16,99,204,205,206].

Of these, the role of AI, Virtual Reality, and augmented reality in developing the online shopping experience has taken center stage in creating a feeling that was not possible before. Using VR, a consumer can be transferred into virtual environments whereby one can explore products as though they were in a physical store. A virtual encounter helps the consumer to make a wiser decision, by getting the chance to observe products in 3D, in real-life surroundings, or even virtually try them on. That is very helpful in cases of clothes, furniture, and home decor, where a physical feel is more important in decisions about selection. VR and AR make virtual shopping more interactive; it also bridges the gap between online and physical retail by creating a much richer and more interactive experience. Just like virtual assistants do—through Amazon Alexa or Google Assistant, online shopping is even easier to use and becomes hands-free. Users can place orders, check the availability of a product, and get personalized recommendations through voice commands only. This will also help people in cases of multitasking or when one has mobility problems, as it is to be used to shop without actually touching a screen. The convenience and easiness of voice-assisted shopping further enables quicker decision-making and faster checkout processes, hence making this process even smoother for the user [3,16,99,192,199,206,207].

Examples of such intelligent devices include networked refrigerators and Smart Home systems. These further enhance convenience by automating aspects of the shopping process [105,106,107,121,122,123,124,132,133,134,135,136]. For example, a refrigerator that is able to monitor its contents automatically reorders groceries when the supply of certain groceries in it starts running low [136,137]. The consumer is thus greatly eased of a lot of time and much hassle. Hence, integrated intelligent technology makes life easier for users by keeping them updated about their grocery requirements without them having to go through the hassle of managing the inventory manually. In the same line of argument, in the future, Smart Home devices will be updating in real time on products, promotions, or delivery statuses for a more connected and dynamic shopping experience [138]. Their potential is not only to upgrade the convenience aspect but also provide customer satisfaction with personalized, efficient, and enjoyable experiences. Consumers feel more connected and in control because of personalized product recommendations, customized promotions, and immersive shopping environments [132,133,139,140]. As a strategy, AI, VR, voice assistants, and other smart devices were applied by the retailers to provide shopping experiences that would meet each customer’s personal needs. Thus, it may assist customers in feeling more aligned with—and bonded to—the retailer. This also helps retailers to be more faster and responsive with changing consumer demands to make the shopping experience exciting and relevant [200,201].

But this would indeed be where the transforming potential lies: Technologies are in constant flux, metamorphosing with each change of imperative from consumers. As AI systems continue to evolve in their sophistication, they learn in real time from user behaviors and build on their suggest-and-improve capabilities with increased knowledge—more often than not, well in advance of the needs arising themselves. Also, as these VR and AR technologies continue to evolve and spread, the virtual shopping experience they will create is bound to only get sharper [3,108,109,111,187,190,191]. Smart devices integrated into the greater retail ecosystem promise a new world of improved interconnectivity and automation in shopping, whereby technology instinctively manages the mundane tasks of shopping and frees the consumer to focus on what they really want [110,111,114,115].

Table 9 summarizes the impact of online shopping technologies on the improvement of the user experience.

Of the technologies currently changing the online shopping environment, perhaps AI holds the greatest, almost boundless, potential for improving the user experience. After all, no other technology or platform can analyze huge swathes of consumer data and make personalized recommendations in real time. Knowledge about how users have behaved on a website, what they like, and what they previously purchased means AI can present very relevant product suggestions and promotions that are likely to be very personal and engaging with shoppers. For this reason, personalization will make buying more effective and agreeable because consumers will be exposed to products in line with their tastes and needs. Therefore, AI-based personalization has a direct consequence on customer satisfaction besides incentives for loyalty and repeat customers [16,99,206,207,208,212,213,214].

Other facets of online shopping will also be refined with the use of AI, from making it easier to find just what customers want to having appropriate inventory levels. AI-powered chatbots and virtual assistants can also provide real-time customer service, day in and day out, from simple queries and navigation down to actually helping the customers complete their purchases. This feature will make online shopping very convenient because it minimizes friction, hence allowing consumers to find quick solutions to problems without having to wait for human interaction. AI provides operational effectiveness for retail through optimization of inventory levels, forecasting demand, and automation of processes to ensure price changes while guaranteeing customer shopping experiences [209,210].

Other promising technologies with huge promises of enhancement of user experience involve augmented and virtual realities, felt most specifically within the field of visualization and interaction with merchandise. Though AR and VR close the gap between online and offline shopping experiences, it is in relation to this particular aspect that both significantly amplify customer interaction with their products in a much more interactive and immersive way. Such would be the case with the potential of AR applications that let customers see precisely how furniture would look in their homes or how clothes will look on them without personally trying them out. This would definitely give more confidence to customers in their choices and may reduce the rate of return. On the other hand, Virtual Reality enables clients to enter online web virtual stores where they can make a 3D view of everything, just like they really would be there. These technologies make the experience of users more interactive and realistic; hence, the satisfaction automatically improves because of more intuitiveness and fun [3,192,193,194,195].

While AI, and especially AR/VR technologies, are immense enhancers of user experience, their mixture bears the greatest potential. Thus, Artificial Intelligence-powered insight into the data will lead applications in AR/VR since these are used to personalize immersive experiences that these technologies will provide. For example, AR can display personalized product recommendations based on user preference, while AI tunes those recommendations in real time, informed by the various ways a user interacts with the virtual environment. Aggregated, such technologies will drive seamless, intuitive, and highly customized shopping experiences that surely create far greater involvement and satisfaction for consumers.

Technologies analyzed in this paper thus come with pragmatic—and perhaps transformative—implications both for e-commerce retailers and their consumers. This was an integrated value chain perspective into how so many opportunities for improvement in technology adoption could yield dividends for a retailer in the form of operational efficiency, customer satisfaction, and competitive advantage. But again, AI, VR, voice assistants, and smart devices could be the other means through which retailers will be able to offer even more personalized and efficient services. Yet all these leading technologies require huge investments in infrastructure, data management, and employee training [116,119,135,136].

That means enabling retailers to apply AI on huge volumes of customer information and trigger events like personalized product suggestions, dynamic pricing, and targeted marketing campaigns. These AI algorithms will provide a granular level of understanding of consumer behavior to the retailer by anticipating demand and hence, optimizing inventory and waste management. This would create the path to more customer engagement and loyalty, as probably, consumers would go back to those platforms, which could offer a personal experience to satiate their needs and wants. This would mean also that processes, such as inventory tracking and customer service, would be automated, which would reduce operating costs by further increasing the efficiency with which retailers could carry out their resources in the most powerful way possible [207,208,210].

Both virtual and augmented reality make much practical sense in those cases of enhancement of some online shopping experience for retailers by allowing them to create an immersive environment where customers would interact with the products in 3D, virtually try items on, and see exactly how merchandise would look on their homes or bodies [204,205,206,207,208,209]. This functionality will bridge the physical-to-online gap in shopping, amenably equipping the consumer to make a better purchase decision, hence reducing the chances of return in case mismatched expectations set in. It is important to note that VR and AR require huge investment in technology development, content creation, and system integration, which may be a burden for minor retailers or those with limited resources [198,199,200,211].

What is more, from the point of view of consumers, online shopping technologies make experiences more convenient and productive, letting personalization in. More importantly, voice assistants finally let customers shop hands-free; they would be able to place orders, check product availability, and even get personalized recommendations—all without much hassle. Thus, the whole process of shopping is faster and much more available for people who lead active lifestyles or have some problems with mobility. Moreover, AI-driven personalization makes consumers see only those products that appeal to their tastes and preferences from past behavior and hence, could save them time and make it more pleasant [71,99,205,206].

Smart devices will increasingly have a great bearing on the shopping habits of consumers. For example, networked refrigerators can track inventories of food and auto-order groceries once supplies start to run low. In this way, it clears the consumer to think about other aspects of their lives with less concern about shopping. It automates the conveniences, bucking the rising trend in home automation, making the process of shopping more cohesive and transparent. Still, there might be potential problems concerning privacy concerns and questions about data security since more significant amount of data could be received from these devices and raise questions during the usage and storage of personal information [108,109,111,140,144].

The revised relation of online and offline shopping might consider practical implications. Such a case is best described by VR and AR since they enable hybrid shopping that will allow customers to enjoy all the benefits both from digital and physical retail. For retailers, it would mean a different way of engaging customers, improving the customer journey. In other words, customers can enjoy the comfort of online shopping while still benefiting from in-store experiences with all their sensory and interactive advantages. But this shift in itself presents new challenges for retailers as a raft of different platforms is integrated and seamless transitions are made between digital and physical touchpoints.

In Table 10 there is a juxtaposition of practical implication of analyzed technologies from the retailer point of view and their impact on consumer behavior and the industry.

While both augmented and Virtual Reality are highly promising, each would generally require a customer to have at their disposal certain devices, such as VR headsets or smartphones with AR functions. This will create a barrier for certain demographics—particularly those that may not have the technology necessary, or who are less adept at working with advanced digital tools. In proliferating the platform, such a technological divide might alienate themselves from the mainstream customer segment, leaving customers—be it those in their older years or low-income groups—behind. This is because, for AI-driven personalized recommendations, a great deal depends upon the quality and quantum of data coming from the consumers [3,192,193,194]. That means a lot of personal information is to be collected and managed by retailers, hence raising a number of issues related to privacy and security. Breaches, cyberattacks, and ethical concerns with the use of consumer data—if proper securities are not in place—remain major barriers toward further deployment of AI technologies in online retail [122,174,175,176,177,178,180,181,182,183,184,187].

Another barrier to consumer adoption relates to the diffidence of consumers in trusting new technologies, especially those with AI-driven personalization and automation [178,179,180]. While exciting for the technology-savvy consumer, the idea of an immersive shopping experience may be daunting or, probably for some, uncomfortable because of the level of technology involved in VR. Each of these makes the rate at which their adoption is growing somewhat slow in certain demography groups, hence limiting their far-reaching impacts within the retail sector.

Besides, logistics also form certain barriers to realizing the potential of these technologies. For instance, e-grocery platforms might now embed AI in ways that, for example, require strong data infrastructure, real-time tracking, and accurate forecasting tools for personalized recommendations to make sense and be effective. All in all, Blockchain for supply chain transparency will also not be successfully implemented without cooperation along the chain and the development of secure systems capable of handling complex and sensitive data. The difficulty in coordinating such technological efforts across diverse stakeholders may make them slower to implement and bludgeon the effectiveness of these technologies in realizing their promises [205,206].

As sustainability and ethical sourcing interest grows, a lot of attention needs to be given to the environmental impact caused by these technologies. Energy consumption from AI data centres, VR systems, and Blockchain networks is huge. Whereas these technologies continue to retain more acceptance, the carbon footprint from their operations can grow, thereby setting a paradox for companies desirous of providing environmentally conscious services. The balance between advanced technologies and sustainability might just prove quite an uphill balance for the future of retailers [99,207,208,209].

Table 11 gives an analysis of some future trends likely to be adopted in online grocery shopping. The table analyzes the technological, behavioral, and logistical transformation that is expected in online grocery shopping to give it a comprehensive look for future trends.

In highly developed parts of the world, where there is high development of technological infrastructure—areas like North America, Western Europe, and parts of East Asia—penetration for AI-driven personalization, VR shopping experiences, and smart devices would be relatively high [215,216]. Typically, these areas boast well-developed connectivity platforms, a wide penetration of smartphones, and an openness on the part of consumers to newer technologies. AI can offer hyper-personalized shopping, VR can create immersive retail environments, and Blockchain can offer supply chain transparency in these contexts. Generally, retailers in such regions will be in a better position to deploy such technologies on a large scale and integrate advanced digital tools into the journey of a consumer much more seamlessly [98,217]. Besides that, consumers in these economies are more likely to be tech-savvy, thus more accepting of such innovations and more active in engaging them. This has become evident in virtual stores, AI-driven recommendation systems, and voice assistants, which have mushroomed in this region as for every e-commerce platform [218].

In the emerging markets, however, things take a different shape altogether. Technologies are not diffused as easily in regions like Sub-Saharan Africa, parts of South Asia, and Latin America. First, they are relatively underdeveloped digital infrastructures [98,219]. High-speed Internet access is often not as available in most parts of the developing world, and even in towns and cities, this access can be spotty or expensive. That makes implementation and good use of these data-intensive technologies, such as AI, VR, and Blockchain, more difficult to apply. In addition, many consumers in developing areas of the world simply lack the required devices that are needed for a seamless user experience given the high-end specifications of smartphones or headsets that allow VR exposure. Such contexts usually dampen the potential of these technologies; many of their consumers may be incapacitated to fully experience the ideal benefits that advanced digital tools can offer [98,217].

Socioeconomic elements also help determine the outcomes of online shopping technologies. Households with higher incomes, particularly in the developed world, will own more devices: smart refrigerators with grocery tracking, voice-activated assistants such as Amazon Alexa, and so on. These increasingly make, or at least facilitate, personalization of grocery shopping possible, making ordering online and inventory smooth. Besides, with greater awareness related to sustainability and social responsibility, consumers in such markets are more likely to adapt Blockchain technology, at the very least, to help them verify the ethical sourcing of products [220,221].

In developing regions or among lower-income households, affordability related to smart devices and access to the internet is one of the key challenges. To these consumers, an AI-driven personalized experience may be farfetched or something beyond their reach. Even in markets where this might be possible, or where VR was available to immerse in shopping journeys, there would be some kind of resistance toward change and possibly a lack of willingness to engage in the process to which costs are relatively high when weighed against the perceived values. Thus, less technologically and economically advanced groups of consumers may be more interested in the basic functions of an online store rather than advanced technological features, thus choosing less elaborate and expensive e-commerce solutions [98,215,216,220,221].

A critical analysis of the results of this paper can be done based on the Technology Acceptance Model (TAM). It basically explains that users accept and use any new technology based on two key factors: perceived usefulness and ease of use [222,223,224]. Online grocery shopping, with the inclusion of new technologies such as Artificial Intelligence, Virtual Reality, and smart devices in it, is bound to be clearly on an uptick along both dimensions.

Perceived usefulness is explicit in the way these technologies improve shopping by making it convenient and personalized [225,226]. Examples of such are AI-enabled systems, which are useful in personalized product suggestions and automated ordering with the use of smart refrigerators to save time and reduce effort spent on managing daily tasks. This again is underlined in the way technologies have smoothened the logistics: faster, flexible delivery options that finally allow consumers to fit grocery shopping into their busy schedules.

Perceived ease of use is reflected in voice assistants and intuitive mobile applications, simplifying online shopping [227,228]. Voice-controlled platforms, such as Amazon Alexa and Google Nest, will let customers make voice orders without the tedium of navigation through websites and applications. Machines that can track inventory automatically and reorder groceries should make household needs management quite easy. Such integration of technologies into recognizable, easy-to-operate interfaces makes their adoption higher, since users find them less intimidating and more aligned with their quotidian routines.

With these technologies becoming increasingly easy to use and functional in nature, the results would be increased adoption. TAM goes a long way in explaining why consumers are willing to abandon the traditional ways of grocery shopping for something more digitized and technologically driven since the innovations discussed in the paper clearly address both the usefulness and ease-of-use factors [224,229]. This model also predicts that future innovations, such as Virtual Reality shopping and Blockchain transparency systems, will be more easily adopted if the practical benefits of continued realizability—the technology remaining accessible and user-friendly—continue [225,226,227].

The Diffusion of Innovation Theory has an important role in understanding how new technologies are diffused within a market. Rogers enumerates several factors that influence the rate of adoption: relative advantage, compatibility, complexity, trialability, and observability [230,231,232]. Using this theory, the recent growth of online grocery services can be explained. Technologies such as dedicated grocery delivery platforms were perceived to have a clear relative advantage over traditional methods of shopping, as this would save lots of time and would be more convenient for people during COVID-19. Also, the compatibility of such services through the integration into lifestyle routines on smart devices and mobile apps increases their pervasiveness in consumer life habits. Trialability, or the relative simplicity and ease with which users can experiment with these platforms, also plays a huge role in the diffusion.

The findings of this paper correspond to the key drivers of the Diffusion of Innovation Theory, especially regarding increasing visibility or observability of such technologies [233,234]. More precisely, with an increasing number of users of online grocery platforms, their experiences are spread among others either by social networks or by word-of-mouth, which in turn accelerates the diffusion process and the wider adoption of these platforms.

The Resource-Based View (RBV) puts forward the view that the competitive advantage of a company is determined by the capability of the company to acquire access to resources and capabilities that are valuable, rare, inimitable, and non-substitutable, and utilize them in an effective manner [235,236,237]. The findings of this paper showcase how the adoption of advanced technologies like Artificial Intelligence, Virtual Reality, and smart devices by grocery retailers is helping these becoming strategic resources in drawing a competitive advantage against an evolving marketplace.

From the RBV perspective, such technologies as AI-driven personalization and smart devices for inventory management automation represent valuable resources and, thus, enable retailers to grant superior customer experiences [238,239]. Predicting consumer preferences, optimizing logistics, and enabling speedier and more convenient services, these technological innovations make the process of enhancing customers’ satisfaction and loyalty more painless [240,241]. Anticipating customer needs and offering customized suggestions, therefore making the process of shopping much speedier and more efficient, is what really differentiates such firms in today’s competitive market that is driven by convenience and personalization in consumer choice [242].

It is observed that the adoption of VR and the metaverse shopping environments signifies a rare as well as an inimitable resource. Not every retailer can design fully immersive virtual shopping experiences; it provides early adopters with distinctive market positioning [237,238,243]. Advanced platforms create an enjoyable, differentiated experience that competitors cannot easily imitate mainly due to the technological and infrastructure costs, which are extremely high when it comes to developing such systems.

Another critical aspect of RBV frameworks refers to inimitability [235,236,237,238]. It is not easy for competitors to imitate the integration of AI and smart technologies into grocery supply chain and customer interaction models. AI systems require huge data sets, sophisticated algorithms, and huge R&D investments. Thus, the firms that have already developed these systems fall into first mover’s advantage, whereby it becomes rather difficult for any new entrants to emulate similar technological sophistication without having to sacrifice much in terms of time and resources.

In fact, the strategic relevance of these resources is further consolidated by their non-substitutability [238,239,240]. Indeed, the functionality of AI, VR, and Smart Home devices is relatively difficult to substitute by the traditional models of grocery shopping. For example, the personal touch of convenience accorded to customers in AI-driven ordering of groceries, or the immersive shopping experience delivered by Virtual Reality, is not easily substitutable by standard e-commerce or physical store formats, and therefore such technologies are irreplaceable for firms that intend to achieve long-term competitive advantages.

## 5. Conclusions

The digital revolution in grocery shopping transformed what could be termed “consumer experience”, mainly because of technological changes and fluctuating expectations among consumers. Considering the radical changes both in-store and online, the main technological innovations that have created a sea of change include the rise of Artificial Intelligence, Virtual Reality, voice assistants, and smart devices. The findings brought out how these technologies make experiences more convenient, personal, and effective. It has really changed the whole manner of interaction between consumers and retailers forever.

While AI-driven platforms and smart devices make mundane tasks easier—for example, a refrigerator that can track inventory and then order groceries sans interference from the consumer—such platforms support personalization in equal measure. Contrasted against this, online grocery platforms have become enviable runs much more recently underpinning advanced logistics and delivery systems in ways that finally meet the demands of immediacy, flexibility, and use that consumers seem to want. Besides, VR and the metaverse built up the retail experience with immersive and interactive environments that bridge the divide between physical and digital retail. Simultaneously, one felt there is a greater emphasis nowadays on sustainability and eco-friendly practices, given the fact that today’s consumers pay so much more attention to transparency and ethical sourcing. Some of this wave of creative responses has targeted these demands: Blockchain, local sourcing, carbon-neutral delivery options. Health and well-being services have joined online grocery platforms offering AI-powered dietary recommendations and personalized nutrition plans. These technologies bring great opportunities in improving the shopping experience. Conversely, they come with a set of challenges that relate largely to issues around accessibility, data privacy, and the possibility of digital exclusion. The potential impacts that they shall have on moral and ethical levels will be most important at the time when future wide applicability brings them about as the development is further unfolded and is driven by the benefits derived from such technological progress.

This paper, therefore, launches into a critical review of the technological changes sweeping across the grocery shopping landscape with recent introductions of Artificial Intelligence, Virtual Reality, and smart devices. Originality can be viewed as firstly, new technologies such as AI, VR, voice assistants, and Blockchain are applied to virtual store settings and also to physical store settings, one by one and in combination, for the first time. Then it introduces VR, or more lately, the metaverse for an immersive shopping experience that is relatively new in retail circles. This paper does not limit itself to academic sources but encompasses updated information on the ongoing practice: current discussions of the use of Blockchain for supply chain transparency and sustainability effort are among many others, thus filling the gap between pure theory and practical innovation. Considering the theoretical contribution this paper will make into the scientific world, the investigated issue of this paper is how convergent technologies jointly influence consumer behavior and retail strategies. With models like the Technology Acceptance Model and the Diffusion of Innovation Theory, further insight is provided into understanding how consumers would adopt new digital tools for grocery shopping. It further contributes to the resource-based view of firms’ discussion by considering how grocery retailers might employ such technologies in a competitive advantage-seeking way. The holistic approach enhances the theoretical framework on digital transformation in retail and prepares support for further research on how changes in technology and consumer behavior interrelate within this rapidly changing sector.

Future research should focus on the adaptation of consumers and their satisfaction with immersive shopping of VR and the metaverse, together with its behavioral implications. The research should focus on how VR affects the shopping experience in terms of both short-term engagement and long-term customer loyalty and should be useful in providing insights into how immersive technology can be tailored to a wide variety of demographics. No less important are studies on ethical implications and privacy concerns of AI-powered personalization. It would also involve researching consumer trust levels regarding AI-driven recommendations and finding out what kinds of data practices contribute to the highest degree of user acceptance, especially in those cases when personalization becomes more and more sophisticated. Further research on voice assistants in retail will better point out how to enhance conversational accuracy for complex orders and refine the user experience for consumers at all different levels of tech-savviness.

In real life, it would be prudent for retailers to implement technologies in online grocery shopping in phases and also for it to be user-centered. Yet another aspect is that while integrating VR and the metaverse, the retailer may want to start a pilot of VR-enabled virtual shopping by controlling either the product categories or allowing only certain sections, as in virtual aisles—those which best mirror layouts from physical stores. The installation of VR headsets or kiosks in selected stores—in partnership with a technology provider—allows customers to try the technology before buying into its online variant. The retailers must also be able to design intuitive VR interfaces that do not require advanced technical knowledge; such a system would ensure that VR shopping is available to the mass of people.

In general, AI-driven personalization needs an extremely high degree of balance between utility and transparency. Recommender engines should change in real time based on browsing behavior, user preference, and past purchases. To gain customer trust, it is important that retailers make it clear what kind of data is collected, provide options for user preferences, and explain how personalization enhancements may prove useful to users. The integration of AI with the available grocery platforms—listing and transition of apps and websites—will be smooth, and recommendations will be highly relevant, especially with regard to the kind of customers who are health-conscious or have some particular dietary restrictions.

Apps and websites now incorporate voice assistants that allow better accessibility to the busy consumer or multitasker. Food retailers need to continue investing in honing conversational accuracy regarding voice-activated ordering, making sure it captures regional language and special grocery terminology. Otherwise, this may be taken a step further by integrating with leading voice assistant platforms such as Amazon Alexa or Google Assistant. It also promotes voice assistant integration as a key feature that could change one’s mindset for reordering staples or even tracking deliveries.

For Smart Home devices—most especially, refrigerators and displays with grocery integration—the focus of the retailer has to be on partnerships with appliance manufacturers. Ensuring that refrigerators can track groceries and allow sharing of data with retailer apps will grant seamless automated ordering to users. The retailer may promote compatible smart devices and incentivize purchases by offering groceries at a discount for people with connected appliances. AI-powered notifications about the need for restocking or an expiration date can avoid food waste and add value to customers by reflecting current sustainable trends.

For augmented reality, retailers need to create user-friendly AR features in mobile apps, enabling them virtually to see any product in real time. Examples include how groceries—perhaps size comparisons—fit into their kitchens, enhancing confidence in that purchase decision. For such a feature, perhaps offer inexpensive means to actually realize interactive augmented reality catalog features in apps providing this experience, especially for high-value items or items that are purchased the most, like produce or packaged goods. A second application is Blockchain for supply chain transparency, aimed at trust-building in product sourcing. With the use of Blockchain, the history of origin, transportation, and processing of every single product is at the disposal of consumers. But taking it one step further and communicating the benefits of this technology, especially to organic, fair trade, and locally sourced products, will be appealing to ethically conscious consumers. Blockchain transparency has the potential to even make product recall processes better, hence making consumers more confident in their choices. The limitations of this paper largely developed from a reliance upon secondary data since a large part of the analysis is from review material currently in existence in terms of literature and industry reports. Conclusively, this gives an overview of what is happening in current technological development in grocery shopping but does not provide original empirical findings to shed light on consumer behavior and how well the technologies at hand work.

Another limitation could be that this paper discusses the technologies from their conceptual and theoretical positions and does not go deeply into what practical challenges may arise when the retailers actually try to apply them. Most of the discussed technologies, such as Artificial Intelligence, Virtual Reality, and Blockchain, require huge investments in infrastructure, training, and further maintenance. The high cost of development, integration, and maintenance may lead to such adoption being a financial or operational barrier for smaller retailers. Besides the financial aspect, there comes the technological know-how. The necessary competencies for retailers to understand and manage such systems themselves mean competent personnel hires or outsourced third-party providers once more, hence raising the cost and complexity of adoption.

It should also be mentioned that a significant limitation of the research was the reliance on various available sources, both literature and the Internet. The authors conducted a review, in which they cited 243 references. However, they focused on reviewing the available sources and did not present a sharply critical approach. Therefore, it is worth noting that another approach to this review article could be a critical analysis of the reviewed sources. This could undoubtedly be the direction of further research and the subject of another article, which will not be typically descriptive but strictly critical.

Other areas that may inform digital grocery shopping in the future are those struggles to understand how emergent technologies will continue to shape consumer behavior, retail strategies, and then market dynamics. In fact, one of the highly promising areas involves the investigation of empirical evidence within the adoption and consumption of Virtual Reality, Artificial Intelligence, and the metaverse for grocery shopping by consumers. While the stages of development for VR shopping are still so new, realistic consumer interactions, the levels of satisfaction, and long-term involvement with VR shopping can offer very useful research through which to learn how to move forward with understanding the realities of its viability and scalability. Another very useful area of future research involves how these technologies might impact data privacy and security. That has involved the collection and processing of personal data by AI and smart gadgets in the process, and research from consumers into trust, regulation challenges, and the ethical consequences of using data have reached such relevance in relation to online grocery shopping. It could also maybe look at how retailers balance personalization and convenience with extensive measures toward data protection. Other probably attractive future research could be directed at delivery logistics, packaging, and waste generally, in the context of environmental sustainability of online grocery shopping. It will also investigate the environmental trade-offs between the convenience of home delivery and the carbon footprint created by more frequent deliveries and how the uptake of eco-friendly packaging innovations and carbon-neutral delivery models by retailers is occurring.

While online grocery shopping technologies, such as supermarket apps, delivery services, VR apps, and smart devices, offer convenience, they also raise concerns. They can create social inequalities (e.g., social exclusion of the elderly) and digital divides. Ethical issues related to data privacy and consumer manipulation are very important. In addition, smaller retailers face significant barriers to adopting these innovations, which can contribute to their problems or even closure. To ensure responsible growth, the industry must balance technological advances with inclusivity, sustainability, and transparency.

## Figures and Tables

**Table 3:** Examples of smart refrigerators used in food ordering.

Name of Technology /Application	Country	Description	References
Samsung Family Hub	United States	Samsung’s Family Hub smart refrigerator comes fitted with a touchscreen from where users can browse for recipes, create shopping lists, and even place orders for groceries using integrated apps such as Amazon Fresh and Walmart. The refrigerator also tracks inventory in real time and notifies users when items are near expiration.	[147,148]
LG InstaView	South Korea	The LG InstaView smart refrigerator has a glass panel inside the device that lights up and makes it much easier to see all its contents. This device also enables cooperation with the LG SmartThinQ app, which enables users to inspect food inventory and get notified of expiration dates, aside from ordering groceries online via cooperation of the said system with various grocery delivery services.	[149,150]
Bosch Home Connect	Germany	Connected via Bosch’s Home Connect technology, the Home Connect app lets users manage their foods’ inventory, receive reminders of products that are near expiration, and even order groceries from local stores. What’s more, the system suggests recipes based on the available ingredients.	[151]
Panasonic Smart Fridge	Japan	Panasonic’s smart refrigerator runs on IoT technology, which makes it possible for real-time monitoring of products. Users can see what is inside their refrigerator through an application, get automatic updates on when any product expires, and even order groceries from various online retailers through the app.	[152,153]
Miele Smart Fridge	Germany	Miele’s smart fridge is integrated with the Miele@home app through which the user can track food, get notifications for food, and even order groceries. It integrates with various grocery delivery services.	[154,155]

Source: Authors’ own work on the basis of: [147,148,149,150,151,152,153,154,155].

**Table 4:** Benefits of using smart refrigerators as food-ordering devices.

Benefit	Characteristic
Convenience	Grocery shopping with smart refrigerators is quite easy. Users can order items directly from the appliance’s interface or on the connected application. This cancels out the need to make a manual list and further go to shop, saving both time and effort.
Time savings	Smart refrigerators save time by automating the management of food supplies—ordering groceries with users’ voices or via a few taps on their display.
Reduced food waste	Expiration notifications and inventory tracking by automation enable users to track food items for use before they expire. Food inventory management, in this respect, ensures that wastes are reduced further to smoothen the operation of a functional, cost-effective kitchen.
Personalized shopping experience	Intelligent refrigerators take advantage of various applications of Artificial Intelligence, such as consumption and preference trend studies, to provide users with personalized shopping suggestions. With this personalization, users can get suggestions and orders in full accord with their needs and dietary requirements.
Enhanced meal planning	Recipe databases, combined with current inventories of what food is available, allow meal planning based on accessible ingredients. This feature further supports enhanced meal planning and reduces the chances of having to go to the grocery store at the last minute.
Improved inventory management	This makes it rather easy for users to manage their inventory, as it can track food items in real time. It can let users know when items are running low, and before they actually run out, users can reorder them to make sure they will always have ingredients in store with them.
Reduced shopping errors	Smart refrigerators automatically generate reorders for repeatedly used items based on the history. This reduces any possibility of forgetting some of the important groceries. The user, in this case, doesn’t need to remember each and every particular item, as it is already dealt with that the supplies will be available to them.
Increased accessibility	Voice control integrated into the digital interface makes it easier for users to use while interacting with their refrigerators for placing orders, which will also not be a hassle without considering the location or physical condition. This can be of great help in cases where people have reduced mobility or very busy lives.
Integration with online platforms	Most smart refrigerators are connected to major online grocery stores dealing with a wide range of deliverable products. Smart refrigerators create an opportunity for the user to enjoy a hassle-free shopping experience in selecting their preferred store and adjusting the delivery accordingly.
Healthier eating choices	Smart refrigerators can provide nutritional information and even suggest recipes that could help in eating healthier. The appliances will truly enable the user to make better choices about food intake and be quite sure their diet is precisely tailored to their health goals and dietary restrictions.

Source: Authors’ own work on the basis of [82,83,84,85,86,87,88,100,101,102,135,136,137,138,139,140,141,142,143,144,145].

**Table 5:** Examples of voice assistant usage in food ordering.

Name of Technology/ Application	Description	References
Amazon Alexa	This voice assistant in the US also provides users with the ability to order food from all the major services, such as Domino’s Pizza and Grubhub. Users can say something like, “Alexa, order a large pepperoni pizza from Domino’s”. Alexa will process the order, process the payment for it, keep updating in real time about the status of the order, and even estimate the time of delivery. This virtual assistant will remember past orders and user preferences. The idea is also integrated into other Smart Home devices to enable users to multitask—for example, appliances in the kitchen until the food arrives.	[157,158,159,160,161,162]
Google Nest	Nest uses Google Assistant for further facilitation in ordering food from services such as Deliveroo or Just Eat, saying something like, “Hey Google, order sushi on Deliveroo”. The Assistant will turn it on to enable the placing of an order and making a payment to update the status of the order about its progress. Integration with a variety of local restaurants and delivery services makes it further comfortable and friendly. Integration with other Smart Home devices is present, too.	[163,164,165,166,167,168]
Apple HomePod	Apple HomePod places food orders using Siri: “Hey Siri, order a burger for me from the nearest restaurant on Menulog”. Further, Siri moves ahead with an ordering application and makes payment while tracking the order status. Siri can be used to recommend restaurants and meals. Similarly, Siri works in the functionality of home devices.	[169,170]

Source: Authors’ own work on the basis of [157,158,159,160,161,162,163,164,165,166,167,168,169,170].

**Table 6:** Data privacy concerns and user acceptance related to smart devices and AI in food ordering.

Aspect	Data Privacy Concerns	User Acceptance Factors	Important Issues
Data collection	Smart devices collect data on nearly everything, from user habits to consumption patterns, health information, and inventory levels.	Users would actually be eager to disclose this information if they saw some added value in doing so: convenience for them, customization, or cost savings.	This would be a sensitive trade-off of convenience against privacy, whereby the user may accept data collection if they see significant value in return.
Data storage and security	The sensitive information kept regarding consumption and personal preferences turns the Smart Home into a potential target for data breaches and misuse of information.	Confidence for the user may increase substantially, provided these intelligent devices handle the data well and explicitly outline rules for handling it.	Therefore, companies should be interested in investing in robust encryption and secure cloud storage. Also, addressing user concerns through clearly communicating their best practices with respect to data protection will help gain confidence among users.
User control over data	Besides this, one can often lose the ability of users to control or understand how data is collected, shared, and used, creating mistrust.	User control and acceptance could be even better if users were given choices in managing their data and adjusting their settings concerning privacy.	Accordingly, customizable privacy settings and transparent data-sharing policies place control with the users, who, in turn, show greater acceptance and trust in smart devices.
Transparency in data use	Moreover, an informal data-use policy may lead to more skepticism in informed ways as to how data will be used or shared with third-party services.	Explain clearly what data usage is for and for what: How AI is going to make recommendations more personalized or shop on behalf of the user.	Demonstrated cases of renowned uses of data and the gains coming thereof—in terms of recommendations, reduced food wastage, or anything to be made by the companies—assure and gain trust from the users.
Third-party access to data	Data-sharing partnerships with third-party vendors, say, grocery delivery services, introduces risks.	In this case, they may be more willing to do it, since they would know such access is strictly controlled and the direct benefit derived is by the users themselves.	The policies for articulating the sharing of data explicitly with third-party platforms should have features like opt-in, enhancing transparency, and enabling users to make more interactive decisions regarding the usage of data sharing.
Impact of AI personalization	AI personalized recommendations—in the absence of clarity on how their data drives that process—come off as invasive to users.	The adoption goes up when the recommendations actually feel useful and relevant, not pointedly targeted or invasive.	Transparency of AI in how it is tailoring things for comfort by the user. Showing users how AI improves shopping is starting to build trust in the use of this technology.
Compliance with regulations	Following regulations such as the GDPR is a key milestone that will ensure that users’ rights are secured with regard to handling and privacy of their data.	Indeed, to note, companies do have strict data-protection rules; due to which, there is increased trust and acceptance of such kind of devices.	Compliance with data-privacy regulations is most often at the core of gaining acceptance from users and offers a safety net that would reassure users that their data was well protected.
Data retention policies	If it is not conveyed when kept, it could introduce the notion of indefinite storage of personal data.	Clearly stated retention periods and transparency of the data increase comfort regarding the collected data as it reduces long-term risks.	What would be comforting, in addition, with regard to data sharing, would be articulation that details the data-retention policies—the kind that prescribes the deletion of such information after some periods of time.

Source: Authors’ own work on the basis of [172,173,175,176,177,178,179,180].

**Table 7:** Comparison between Virtual Reality (VR) shopping experiences and traditional online shopping.

Aspect	VR Shopping Experiences	Traditional Online Shopping
User experience	Immersive and interactive, wherein users would navigate virtual aisles, view 3D products, and get a sense of presence similar to physical stores.	Visual and pretty straightforward: two-dimensional pictures, with a description of the goods in text form on any website or application.
Product interaction	It enables the customer to have an opportunity to explore products in a three-dimensional environment. This makes interaction with the product more efficient because they can view them from a number of dimensions.	The limitation is that it allows only two dimensions—images and descriptions—and it does not allow tangible interaction with products.
Personalization	It is highly capable of personalized, immersive experiences where VR responds to user behavior and preferences while working in virtual worlds.	It does enable personalization through recommendation algorithms.
Convenience	In VR, users could shop at home but have to wear VR equipment; this could be limiting.	Highly accessible: It requires only a smartphone, a computer, or a tablet with access to the internet.
Social features	Social shopping experiences: users can go shopping virtually with friends or work with virtual assistants.	Only very basic social features exist, such as a shared shopping list or online chat for customer support.
Customer engagement	More engaging due to the immersive nature; virtual environments expand user attention and interest in products.	Standard engagement: This would generally depend upon the architecture of the website along with its interactive features, like videos and product reviews.
Technology requirement	Requires VR headsets and compatible devices, along with high-speed internet for the best experience, which may not be accessible by all consumers.	It requires only a normal internet and device to operate; therefore, it is more accessible.
Purchase-decision process	Provides an environment somewhat similar to in-store shopping, which can decrease hesitation to purchase by allowing users to “see” products in a realistic environment.	The customer reviews, product descriptions, and images an e-commerce mainly depend upon, especially for a more sophisticated product, do not give that much information.
Cost implication	Business issues: Higher up-front costs to develop VR environments; the consumer must have VR equipment.	Cheaper for the enterprise in creating and maintaining a website. On the customer’s side, no specialty hardware is needed other than common digital devices.
Sustainability	Perhaps sustainable, as this will potentially reduce the number of physical store visits; however, the manufacturing and use of VR technology are harmful to the environment.	Reduces the amount of travel to and from stores, reducing transportation emissions. It “may be problematic on account of high data consumption because it streams product videos and images which in turn are most likely to have impacts on the environment”.
Emotional appeal	High emotional engagement due to the sensory-rich environment that VR creates; this would build up a sense of adventure and thrill.	Weaker emotional activation: more utilitarian, traditional online way of shopping devoid of immersive sensory appeal.
Future potential	Emerging yet fast-growing, thus opening avenues with the metaverse for integration and features of social interaction, even as access is confined to a few early adopters.	Mature, widely adopted technology that has been optimized and continues to be optimized, but it does not offer immersive and social interaction features.

Source: Authors’ own work on the basis of [3,70,187,190,191,192,193,194,199].

**Table 8:** Effectiveness, advantages, and disadvantages of online grocery shopping technologies.

Technology	Effectiveness	Advantages	Disadvantages
Virtual Reality shopping	Immersive experience replicates physical shopping by letting users view products in 3D and interact with them as they would in a store.	Offers highly engaging, true-to-life shopping experiences to shop socially in a virtual space. There is a greater interaction with the product.	High initial costs for VR development and equipment. Requires advanced hardware and fast internet. Limited accessibility.
Metaverse integration	Amplifies VR by allowing users to shop in both collaborative and social ways: combines online and in-store experiences within a continuous virtual space.	Social interaction with others through avatars in an interactive virtual experience. Realistic simulations of stores. Personalization of shopping environments.	High development and maintenance costs. Accessibility barriers due to VR requirements. Privacy and data security concerns.
Augmented reality for product interaction	It is good for showing product details, visualizing how the products fit in the home, or offering try-before-you-buy options.	It enhances the visualization of a product. It allows interaction with the details of products. Helps in food items where packaging needs to be considered.	Limited use cases in grocery (more effective for non-food items). Requires device compatibility. Costly for retailers to implement.
Smart devices	Offers automation to grocery ordering and inventory management, thus offering convenience by integrating it within grocery apps and AI-driven suggestions.	Reduced food wastage with the help of expired notifications. It enhance inventory management. Personalized automated shopping recommendations.	High initial cost for devices. Privacy concerns due to data collection. Limited to users with compatible Smart Home ecosystems.
Voice assistants (e.g., Alexa, Google Assistant)	It enables hands-free, convenient shopping: a user can order groceries or meals by giving simple voice commands.	Easy to operate, especially by busy households. It allows reordering and tracking with no manual effort. It improves access.	Limited conversational accuracy for complex orders. Privacy concerns with voice data. Restricted integration with certain vendors.
Blockchain for supply chain transparency	Effective in building transparency and trust in food sourcing; it can enable the tracing of the origin of products and their certifications.	Enhances traceability and accountability in supply chains. Reduces risk of fraud and mislabeling. Supports ethical sourcing.	High cost and complexity of implementation. Limited consumer understanding of Blockchain. Requires extensive industry adoption.

Source: Author’s own analysis.

**Table 9:** The potential of online shopping technologies to improve the user experience.

Technology	How It Improves the User Experience	Key Benefits
Virtual Reality shopping	Offers immersive 3D shopping experiences much like real-life stores, where users will be enabled to navigate virtual aisles and interact with products.	Offers realistic shopping environment similar to in-store experiences. Enhances product visualization and confidence. Potential for social shopping with friends/family in virtual space.
Metaverse integration	This further extends VR experiences through social and interactive shopping: All users shop with other people in a virtual environment.	Encourages social engagement in online shopping. Customizable virtual shopping spaces. Creates a unique, engaging experience.
Augmented reality for product interaction	Allows the user to view the details of products that are in space, such as how big something fits at home.	Improves product confidence before purchase. Useful for trying before buying, particularly with complex packaging or multi-use products.
Smart Home devices	Automates grocery management by keeping an inventory, sending notifications of when things expire, and suggesting recipes based on ingredients on hand.	Reduces food waste through automatic expiration alerts. Streamlines inventory management. Enhances convenience by automatically reordering essentials.
Voice assistants	It enables hands-free voice shopping where customers can add items, reorder, or check status with their voice. It offers trackable products throughout the supply chain, with verified authenticity and sources ethically.	Highly accessible and convenient for busy or multitasking users. Easy to use, particularly for families and individuals with mobility limitations. Integrated with Smart Home ecosystems for seamless interaction.
Blockchain for supply chain transparency	Offers immersive, 3D shopping experiences, truly emulating stores in real life; users will be able to navigate virtual aisles and interact with products.	Increases trust and confidence in product provenance. Reduces fraud and counterfeiting. Promotes ethical practices and sustainable sourcing. Enhances consumer awareness and product accountability.

Source: Author’s own analysis.

**Table 10:** Practical implications of online shopping technology on retailers and consumers.

Technology	Practical Implications for Retailers	Implementation Strategies for Retailers	Impact on Consumer Behavior and the Grocery Industry
Virtual Reality shopping	It can offer immersive shopping experiences, enabling retailers to offer different services and interactively engage with consumers.	Invest in VR-ready platforms and VR content that can emulate the experience of being in a store. Offer in-store VR headsets or kiosks to trial before going full-scale online.	Consumers will have more interactive shopping and may become loyal to the stores offering such VR options. VR can reduce return rates since customers would be making more assured purchases.
Metaverse integration	Offers opportunities for social shopping and brand community engagement, helping the retailer establish a very strong brand identity.	Branded, customizable metaverse spaces to build community. Switch on social shopping functionality that allows friends to shop together online.	More consumer engagement and brand loyalty. The grocery business may become more social, community-oriented shopping.
Augmented reality for product interaction	Using AR allows customers to see detailed products that may improve purchase confidence and lower returns.	Build AR-enabled product catalogues; try-before-you-buy options. Provide interactive AR in apps to see and better understand products.	Enhances confidence in purchasing and lowers return rates. Consumers may engage more with products they can “see” and “try” in their own space.
Smart Home devices	Inventory management automation will be possible to prevent stockouts and allow regular purchases.	Collaborate with appliance manufacturers to ensure compatibility. In-app integrations develop the capability for automated reorder when an item runs low.	Promotes convenience and saves consumers from nuisances such as stockouts. Increased frequency of automatic shopping, increasing sales.
Voice assistants	Voice assistants can enable hands-free, convenience-driven shopping. This improves accessibility and simplifies the purchase process.	Voice assistant integrations across apps and websites. Develop voice-activated shopping lists and reorder mechanisms.	Consumers would shop using only voice commands with less hassle, hence increasing impulse buying. In the grocery business, more automation of shopping can occur mainly for repeated needs.
Blockchain for supply chain transparency	Provides traceability in product sourcing and ethical standards, which engenders a lot of consumer trust and makes a brand credible.	Utilize Blockchain for supply chain tracking in a more transparent manner. Educate consumers about product origin with in-app information.	Consumers, especially in organic or local products, may favor transparent sourcing. The industry may see its trend towards transparency in sourcing as competitive.

Source: Author’s own analysis.

**Table 11:** Future trends—online grocery shopping.

Trend	Description
Increased use of Artificial Intelligence	It is envisioned that with deep analysis of consumer data powered by advanced AI, learning from shopping patterns, and hyper-personalization, shopping will be really common. It will predict what users need based on past purchases, propose new products, and allow for the creation of automated shopping lists to dynamically update as preferences or behaviors change. Beyond this, AI will optimize logistics and supply chain management, reducing delivery times while minimizing food waste due to better demand management. Grocery retailers will interact with their customers using AI-powered chatbots, thus having immediate support or solution time.
Virtual Reality and metaverse integration	Immersive, interactive environments will be where the customer experience will shift as VR and metaverse platforms will be further integrated into online grocery shopping. This is about the time when shoppers put on Virtual Reality headsets, walk around digital stories, pick up products off virtual shelves, examine them in 3D, and interact with digital avatars of customer service representatives. The virtual store can appear like a real supermarket, or a completely new, personalized shopping environment can be created. Besides, the metaverse could very well allow for social shopping: One can shop with friends or family members virtually, side by side in a virtual space, sharing experiences from remotely dispersed locations.
Expansion of smart devices in grocery ordering	Smart appliances, particularly AI-powered refrigerators, will undoubtedly be more and more at the forefront of managing groceries. With embedded cameras and sensors, these refrigerators would be able to scan and monitor food items for real-time data related to stock levels and expiration dates. Smart fridges will integrate into online grocery platforms that will automatically trigger an order for items running low to make sure the household is fully stocked with essentials. Smart Home assistants will go the extra mile and allow voice commands to check inventory in the fridge, request the addition of new items, or suggest meals based on what is in the fridge.
Sustainability and eco-friendly practices	Online grocery platforms will further adopt greener practices due to shopper demand for more environmentally conscious ways of buying food. Retailers will provide environmentally friendly packaging with biodegradable or reusable bags and minimize plastic use in delivering products. Moreover, such platforms will focus on local and organic food contributors. This will enhance the reduction of food miles and supports small, regional producers. Innovations in carbon-neutral delivery options, using electric vehicles or drones, will also become more common, enabling consumers to minimize their environmental impact. Food technologies that reduce waste will also be introduced, where AI will suggest recipes using expiring products.
Faster delivery with instant and scheduled options	With increased competition online for groceries, the demand for quicker and more flexible delivery will grow. Online grocery retailers will offer everything from on-demand instant delivery—15−30 min—to more scheduled, precise delivery, allowing a customer to choose an exact time slot for convenience. This will be enabled through a trend in the development of AI-driven route optimization that makes logistics speedier and more efficient. Moreover, the proliferation of “dark stores”—retail locations set up specifically to fulfill online orders—and micro-fulfillment centers will continue to drive delivery times down and bring groceries even closer to urban consumers.
Voice-assisted shopping	Voice assistants, including Amazon Alexa, Google Assistant, and Apple’s Siri, will play a greater role in acting as interfaces consumers use to manage their grocery shopping. With these devices, users will add to their carts, reorder items, or track their deliveries using only their voice, since the process is to be much freer from hands-on usage and consequently easier. In time, voice assistants will be further integrated with Smart Home systems allowing real-time communication between home devices and grocery platforms, which again would make this process even simpler. For instance, the smart fridge can update the assistant to put missing items onto the shopping list.
Subscription-based grocery services	Subscription models will start to be more prevalent: the way to offer consumers consistent, worry-free grocery replenishment. Using AI, those services could learn from consumer needs and enable recurring orders of staple goods like milk, bread, and vegetables when a particular consumer would need them. The implication is that this model reduces manual shopping and ensures the presence of necessities at all times. Subscription boxes also will be designed and personalized based on dietary needs or preferences, and they will find their way to consumer doorsteps at periodic intervals.
Blockchain for supply chain transparency	Blockchain in the grocery supply chain is going to be used increasingly in tracing and authenticity. By means of a decentralized ledger, consumers can trace food products to their very origin for assurance of due care regarding ethical, organic, or other specific standards of sustainability. The result will be more trust by consumers in retailers—most especially for fresh and organic produce. It will also help reduce fraud in food and enhance efficiency in product recall, since everything at each step in the supply chain is indelibly recorded and available.
Integration of health and nutrition services	Online grocery shopping in the future will be further integrated with services guaranteeing health and wellness. AI-driven platforms, while analyzing users’ health information like dietary preferences, allergies, or fitness goals, will offer personalized grocery suggestions. It can integrate well with wearable health devices or apps to enable grocery shopping more holistically, putting wellness first. For example, users who track their intake of calories or monitor for certain nutrients will get automated food suggestions that match their health goals. These integrations will make healthier eating easier and more targeted to the needs of the individual.
Omnichannel retailing	In the future, omnichannel retailing will blur the line between physical and digital shopping experiences. Retailers will move seamlessly between online and offline experiences, enabling customers to shop online and pick up in-store, or to browse physical stores and have purchases delivered. Mobile apps, AR in physical stores, and unified loyalty programs facilitate channel switching by consumers as they choose what best serves their needs at any given moment in time. It will also meet the emerging demand for flexibility and customized retail experiences through this hybrid model.

Source: Authors’ own work on the basis of: [3,50,51,52,53,59,61,62,63,64,65,70,76,77,79,89,92,93,94,95,96,98,99].

## Data Availability

No new data were created or analyzed in this study. Data sharing is not applicable to this article.
